# Very- and ultra-long-period seismic signals prior to and during caldera formation on La Réunion Island

**DOI:** 10.1038/s41598-019-44439-1

**Published:** 2019-05-30

**Authors:** F. R. Fontaine, G. Roult, B. Hejrani, L. Michon, V. Ferrazzini, G. Barruol, H. Tkalčić, A. Di Muro, A. Peltier, D. Reymond, T. Staudacher, F. Massin

**Affiliations:** 1 Université de Paris, Institut de physique du globe de Paris, CNRS, F-75005 Paris, France; 2 Université de La Réunion, Laboratoire GéoSciences Réunion, F-97744 Saint Denis, France; 3 Research School of Earth Sciences, The Australian National University, Canberra, ACT 2601 Australia; 4 Observatoire volcanologique du Piton de la Fournaise, Institut de physique du globe de Paris, F-97418 La Plaine des Cafres, France; 5 CEA/DASE/Laboratoire de Géophysique, Commissariat à l’Energie Atomique, BP 640, 98713 Papeete, Tahiti French Polynesia; 6 Swiss Seismological Service, ETH Zurich, Sonneggstrasse 5, CH-8092 Zurich, Switzerland

**Keywords:** Natural hazards, Seismology, Volcanology

## Abstract

Early detection of the onset of a caldera collapse can provide crucial information to understand their formation and thus to minimize risks for the nearby population and visitors. Here, we analyse the 2007 caldera collapse of Piton de la Fournaise on La Réunion Island recorded by a broadband seismic station. We show that this instrument recorded ultra-long period (ULP) signals with frequencies in the range (0.003–0.01 Hz) accompanied by very-long period (VLP) signals (between 0.02 and 0.50 Hz) prior to and during the caldera formation suggesting it is possible to detect the beginning of the collapse at depth and anticipate its surface formation. Interestingly, VLP wave packets with a similar duration of 20 s are identified prior to and during the caldera formation. We propose that these events could result from repeating piston-like successive collapses occurring through a ring-fault structure surrounding a magma reservoir from the following arguments: the source mechanism from the main collapse, the observations of slow source processes as well as observations from the field and the characteristic ring-fault seismicity.

## Introduction

Caldera collapses are rare (only seven events over the last 100 years) and particularly destructive volcanic events that can induce catastrophic changes in the shape of a volcanic edifice and its environment^1^. Identifying the occurrence of the first collapse at depth is of major importance in evaluating the triggering factors and anticipating the caldera surface formation. This detection can help to predict or at least indicate early future caldera collapses and subsequent consequences such as explosive eruptions, (e.g. those that followed the major Kīlauea Caldera collapse in 1470–1510^2^), or atmospheric impacts^3^.

In the past two decades only four caldera collapses have been monitored by dense geophysical networks: chronologically, the 2000 Miyake-jima, Japan; the 2007 Piton de la Fournaise, La Réunion/France; the 2014–2015 Bárðarbunga, Iceland; and the 2018 Kīlauea, Hawai‘i^4–7^. Laboratory experiments and numerical analyses predicted the occurrence of precursory collapses at depth before the onset of the surface subsidence^8–12^ but observations obtained from adequate broadband seismometers were lacking. Until the present study, the Miyake-jima caldera formation was the only known case with evidence of such deep collapses before the faults reached the surface. The deep collapses were suggested from observations of VLP seismic pulses of 20 s width^13^. However, the timing of the first collapse at depth was not reported. VLP signals observed at volcanoes are generally considered having frequencies between 0.01–0.5 Hz (ref.^14^) and until the 2000 Miyake-jima event, they were generally considered to result from inertial forces associated with changes in the flow of magma and gases through conduits^14^. The VLP signals detected during the Miyake-jima caldera formation were explained by different physical mechanisms: (*i*) a buried geyser model^15^, (*ii*) a piston-like model^4,13,16^, and (*iii*) ring-faulting mechanisms related to shear failure on curved or cone-shaped fault structures^17,18^. 46 step-like tilt changes (TC) were observed during the Miyake-jima caldera formation and among them 39 were accompanied by the VLP seismic signals^4,19,20^. The origin of these TC associated with the VLP pulses have been attributed to either a piston model with a vertical rock column intermittently sinking into a magma reservoir^4,21^ or to a magma sheet model with a large sill-like magma reservoir expanding cyclically^19,20^. Step-like tilt changes accompanied by VLP seismic signals were only reported during and not prior to the 2000 Miyake-jima collapse^21^. Here, we report for the first-time, ULP seismic signals accompanied by VLP seismic signals associated to precursory collapse at depth. We show the possibility of constraining the timing of the onset of the first collapse at depth and that the 2007 Piton de la Fournaise Caldera collapse may correspond to a piston-like collapse occurring through a ring-fault system.

Laboratory-analogue experiments show for roof aspect ratio (i.e. roof depth/piston diameter) between 2 and 4.5 that surface subsidence occurs concurrently with collapse at depth^8^. An important challenge for understanding and constraining the triggering factors of caldera formation in this range of roof aspect ratio (i.e. 2–4.5) is to detect the first collapse at depth before the surface subsidence^8^. This should improve volcano early-warning systems. In the case of the 2007 Piton de la Fournaise collapse, the aspect ratio is around 1.7/0.4 = 4.25 assuming a fault surface approximated by a cylinder with a height of 1.7 km and radius of 0.2 km. The radius is derived from photographs obtained just after the main collapse^5^ and from the extension of the seismicity at depth^22^. The height is estimated from the location of the shallowest compression compensated linear vector dipole (C-CLVD) earthquakes^22^. Interestingly, the roof aspect ratio was also high (between 1.9 and 3.8) for the 2000 caldera formation at Miyake-jima^23^. The historical Dolomieu Caldera collapse, which occurred at Piton de la Fournaise in April 2007, is an unusual case with records from a broadband seismic station and a dense, continuous monitoring network of geodetic and short-period seismic stations. It provides a rare opportunity to study the processes related to early steps of caldera formation.

**Figure 1:**
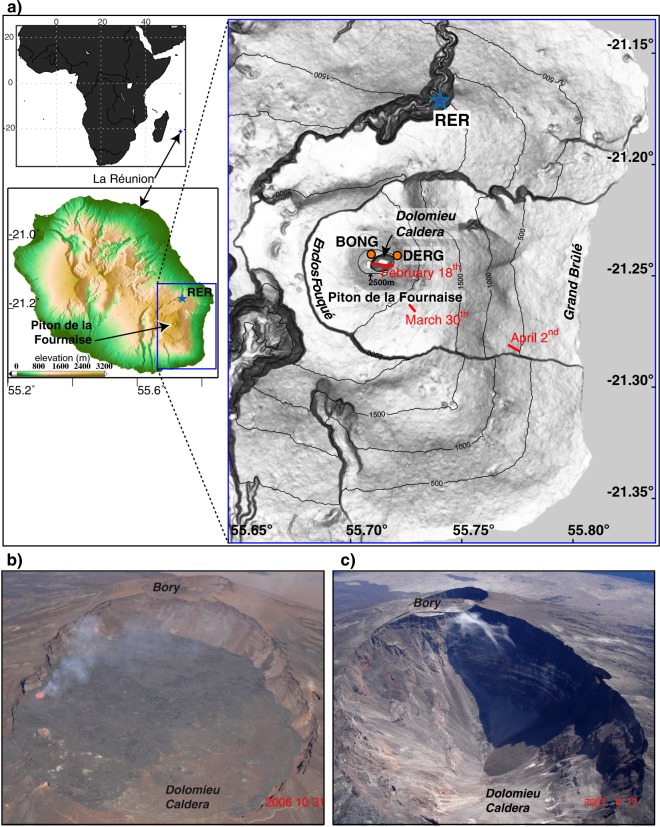
Location of the Dolomieu Caldera. (**a**) The caldera is located on the Piton de la Fournaise massif, on the southeastern part of La Réunion. The broadband seismic station RER is indicated by a star. BONG and DERG are permanent GPS receivers. Fissures corresponding to the 2007 eruptions are also indicated. The map of La Réunion and Africa was realized with MatLab software version R2016a and the map of La Réunion Island was performed with GMT software^72^ version 4.5.7 and digital elevation model from the Shuttle Radar Topography Mission (http://srtm.csi.cgiar.org/SELECTION/inputCoord.asp, 90-m resolution). The SURFER version 10 software (http://www.goldensoftware.com/products/surfer) was used to create the map of Piton de la Fournaise with the digital elevation model of the IGN (http://professionnels.ign.fr/bdalti, 25-m resolution). Photographs of the Dolomieu Caldera were obtained from the Observatoire Volcanologique du Piton de la Fournaise (OVPF) (**b**) before the collapse on October 31, 2006 and (**c**) on April 17, 2007. From April 10, the geometry of the caldera structure did not change significantly^5^ compared to the previous collapses.

Piton de la Fournaise is a basaltic shield volcano built on the southeastern part of La Réunion Island (Fig. 1), in the western Indian Ocean. Its summit cone is located in the old Enclos Fouqué caldera and has two summit depressions: the Bory Crater in the west and the Dolomieu Caldera (about 1 km in diameter) in the east (Fig. 1). In 2006, after a period of intense volcanic activity, the August eruption (August 30 to December 31, 2006) completely filled the Dolomieu Caldera and caused its lower, eastern border (2480 m of elevation) to overflow. In 2007, three eruption phases occurred sequentially: on February 18–19, March 30–31, followed by renewed eruptive activity on April 2-May 1. These eruptive events were associated with a single inflation-deflation cycle starting in January 2007^24^. A low-elevation fissure opening, possibly triggered by deep magma input^24^ started on April 2 at 6:00 (Universal Time), only three days before the Dolomieu Caldera collapse. From March 31 to April 2 a lateral magma migration occurred at depth toward the April eruptive vent. The lateral magma migration from the March 30 eruptive fissure to the April 2 eruptive fissure was suggested from GPS observations^25^. A single lateral magma migration was also proposed^24^ based on the kinematic continuity since March 30 highlighted by the tilt data, combined with the interferometric data showing a large flank deformation area connecting both March 30 and April 2 eruptive fissures^26^. A significant increase of seismicity below the Dolomieu summit and beneath the eastern slope of Piton de la Fournaise was observed from the short-period seismic network from March 31 to April 2 with several thousands of volcano tectonic events^22^. From April 1 at 13:20 to April 4 at 15:44, the seismic crisis was dominated by 315 C-CLVD earthquakes located beneath the summit between the sea level and 0.8 km above. The C-CLVD earthquakes were associated with fluid motion from vertical fluid-filled cracks to a magmatic reservoir^22^. These events were not coincident with ULP signals accompanied by VLP signals as reported in this study for later precursory collapse events (E2 to E5) occurring on April 5 (Supplementary Table 1). On April 5 at 20:48, the Dolomieu Caldera collapsed (event CE1) at the beginning of one of the largest historical eruptions of the volcano^5^. In this study, we report 48 collapse events relating to this incremental caldera collapse. The eruption ended on May 1 at 20:00. A surface subsidence of 340 ± 15 m was estimated four days after the main collapse event from distance measurements using a Leica telemeter at about 20 sites around the caldera^27^. The total volume of lava emitted and intruded during the whole April eruption was estimated as 2.4  × 10^8^ m^3^ (ref.^28^) and the collapse episode led to the development of a caldera volume of 9 × 10^7^ m^3^ (ref.^27^).

## Results

### Data

We used the seismic station RER of the GEOSCOPE seismological network installed in a 4.7 km-long tunnel, 8.2 km north of the summit (Fig. 1). In 2007, this permanent station was the only broadband seismological station installed on La Réunion Island able to record the caldera collapse. The station was equipped with three 1-component STS-1 seismometers^29^ and a Quanterra Q330 digitiser. The three seismometers recorded the three seismic components (vertical, north-south and east-west) of ground velocity. The corresponding instrumental responses of the broadband (BH) raw seismic channels (sampling rate of 20 Hz) are flat in velocity in the 1/360–5 Hz frequency range. Data from teleseismic stations of the global seismic network were also used to determine the moment tensor solution for the main collapse event (CE1) and for surface-wave magnitude estimates.

### VLP and ULP seismic signals associated with caldera collapses

VLP signals displayed dominant frequencies of 0.14 Hz, 0.3 Hz and 0.4–0.5 Hz (Supplementary Fig. 3c). The spectra of Supplementary Fig. 3c suggest that there is a continuous signal from 0.2 Hz down to around 0.02 Hz. In this study, this observation motivated our analysis of VLP signals in the range [0.02–0.2] Hz. ULP signals are characterized by frequencies in the range 0.003–0.01 Hz (Supplementary Fig. 3c) as an increase of the signal amplitude is observed below around 0.01 Hz up to 0.003 Hz. Frequencies lower than 0.003 Hz are not represented to avoid possible problem of STS-1 sensors described near 1/360 Hz in the Global Seismographic Network^30^.

**Figure 2:**
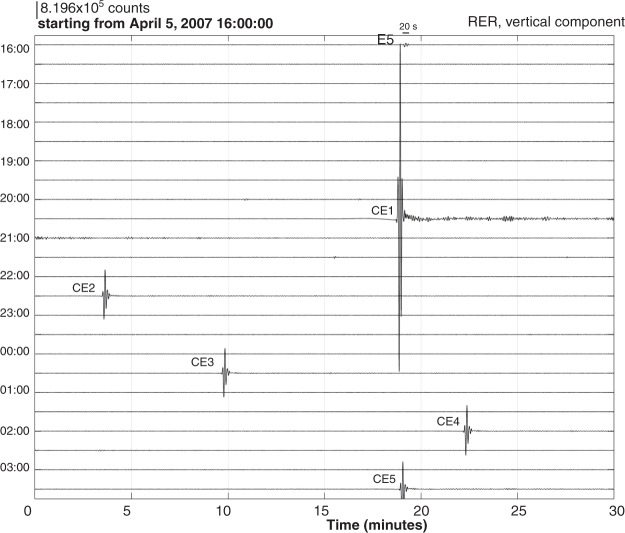
Comparison of VLP seismic signals prior to and during the caldera formation. VLP wave packet with a width of around 20 s is repeatedly observed at RER seismic station during the caldera formation. The seismogram is the vertical component of velocity after: (*i*) removing the mean and the trend, (*ii*) applying a 5% Hanning taper, and (*iii*) band-pass filtering between 0.005 and 0.2 Hz with a zero-phase 4-pole Butterworth filter.

The duration of VLP wave packets (consisting of several oscillations with modulating amplitude) were identified from the following procedure: (i) removing the mean signal, (ii) removing the trend, (iii) applying a 5% Hanning taper, and (iv) bandpass filtering between 0.005 and 0.2 Hz with a zero-phase 4-pole Butterworth filter. We use this low corner frequency to investigate if the duration of VLP wave packets reached around 30–65 s during the caldera collapse episode, as observed during the Miyake-jima Caldera collapse^16^. VLP seismic wave packets with a duration of about 20 s were observed prior to (Supplementary Figs 1a and 2a,b) and during the caldera collapse (Fig. 2). Between April 5 at 20:48 and April 14, seismic records from the RER seismic station clearly show the occurrence of systematic cyclic variation in the signal due to the occurrence of VLP wave packets of about 20 s observed for each of the collapse events over more than nine days (Supplementary Fig. 2a,b).

The intervals between two consecutive events decreased from about two hours at the beginning of the sequence (on April 5, 20:48) to 30 minutes on April 6 (Supplementary Table 1), and then increased on April 7 up to four hours, and to around 37 hours on April 14. Some 48 distinct collapse events (named CE1 – CE48) were synchronous with the caldera collapse and show clear ULP seismic signals accompanied by VLP seismic signals (Supplementary Table 1) during each individual collapse event (Supplementary Figs 3a and 4). Similarly, four events (E2 to E5) recorded prior to the caldera collapse CE1 event are characterised by ULP seismic signals accompanied by VLP seismic signals but these events were not associated with any surface rupture. Events spanning from E2 to CE48 are interpreted as single collapse events (and earthquakes).

### Calculations of event magnitudes

We estimated the relative size of the collapse events from CE1 to CE48 using the surface-wave magnitude *M*_*S*_ and an indirect method similar to the 1968 Fernandina Caldera collapse^31^. The magnitudes of these earthquakes provide the possibility to constrain the evolution of both the seismic moment and the seismic energy release during the caldera formation, but it also provides a way for investigating the possible rupture process. In this study, the moment magnitude *Mw* together with the seismic moment *M*_*0*_ were determined only from the moment tensor inversion for the CE1 event. For the other 47 events, we determined the *M*_*S*_ values, based on the amplitude of surface waves, which does not suffer from saturation at these magnitudes and which allowed us to quantify and compare most of the individual collapse events on a uniform basis. We computed the *M*_*S*_ values (see Methods) from data recorded at teleseismic stations SUR (Sutherland, South Africa), BOSA (Boshof, South Africa), LBTB (Lobatse, Botswana), LSZ (Lusaka, Zambia), KMBO (Kilima Mbogo, Kenya), FURI (Mt. Furi, Ethiopia), LSA (Lhasa, Tibet, China), CRZF (Ile de la Possession-Crozet Islands, France), DBIC (Dimbokro, Cote d’Ivoire), KMI (Kunming, Yunnan Province, China), MBAR (Mbarara, Uganda) and TSUM (Tsumeb, Namibia) for 42 collapse events occurring between April 5 at 20:48 and April 14 as six other collapse events records were contaminated with interference from teleseisms.

**Figure 3:**
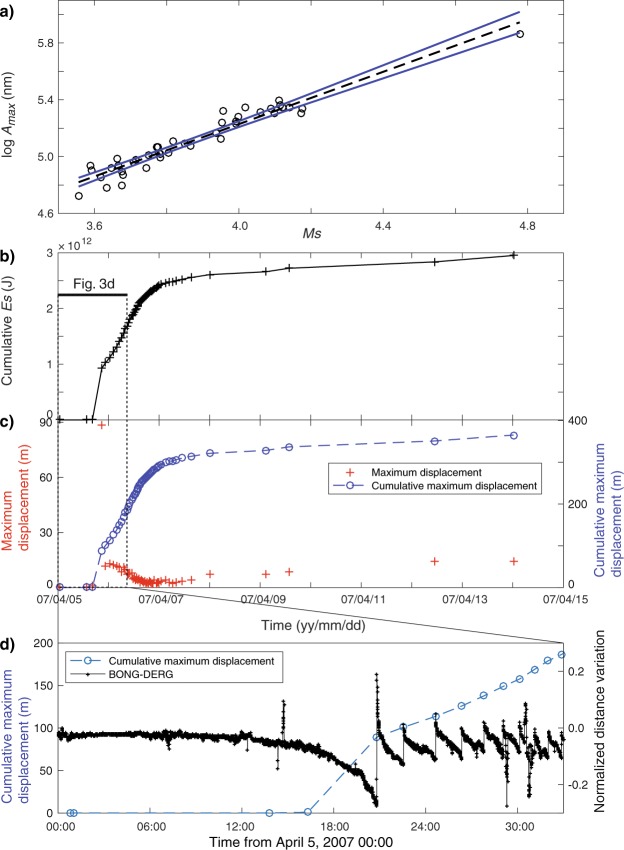
Energy release and displacement associated with each collapse. (**a**) The open circles show values of the maximum VLP seismic amplitude (from the N-S component band-pass filtered between 0.02 and 0.2 Hz) recorded at RER and plotted against the surface wave magnitude *M*_*S*_. The straight dashed line is the least squares fit of this data and was used to estimate *M*_*S*_ for the other collapse events recorded at RER. Blue lines indicate the 95% confidence interval. (**b**) Representation of the cumulative energy release versus time. A box shows the time span from (**d**). (**c**) The crosses represent the maximum apparent displacement computed for each caldera collapse from the seismic moment *M*_*0*_. *M*_*0*_ was estimated from the *M*_*S*_ value. The cumulative displacement versus time is represented by open circles. (**d**) The cumulative displacement and GPS data variations in east-west distance between the BONG and DERG GPS receivers versus time.

The estimated *M*_*S*_ values are between 3.6 and 4.8 (Supplementary Table 1). The uncertainty of *M*_*S*_ for each collapse event is estimated from the standard deviation *σ* of *M*_*S*_ values obtained for each collapse event using multiple seismic station. We had to use an indirect method to estimate the magnitude of the remaining events. We determined the maximum VLP seismic amplitude from the N-S component of the RER seismic station on La Réunion for each collapse event from CE1 to CE48. A good correlation is observed between the maximum VLP seismic amplitude *A*_*m*_ from the N-S component and *M*_*S*_ with a correlation coefficient around 0.97 (Fig. 3a). Therefore, we determined a linear relationship between the two parameters to estimate the magnitude for the other collapse events and smaller events recorded only at the RER seismic station. The resulting relationship is:

1 $${\mathrm{log}}_{10}{A}_{m}=(0.92\pm 0.08){M}_{S}+(1.55\pm 0.31),$$

where *A*_*m*_ is the maximum VLP seismic amplitude from the N-S component of RER seismic station band-pass filtered between 0.02 and 0.2 Hz, (0.92 ± 0.08) is the 95% confidence interval for the slope and (1.55 ± 0.31) is the 95% confidence interval for the intercept.

The seismic energy release (*E*) of all collapse events and precursory collapse events is estimated using the *M*_*S*_ values and the formula^32^ used for the 1968 caldera collapse of Fernandina in the Galápagos archipelago^31^. The energy release rate is nearly constant from about 20:48 on April 5 to April 7 (Fig. 3b). Most of the energy is released during this period. The cumulative energy for the caldera collapse episode and precursory collapse events (Fig. 3b) is 3.0 × 10^12^ J. It is around 25 times lower than the cumulative energy estimated for the caldera collapse of Fernandina^31^.

### Similarity of VLP and ULP seismic signals

Individual VLP seismic wave packets related to the Dolomieu Caldera collapse show similar waveforms prior to and during the caldera formation (Supplementary Fig. 2a,b). They were determined from the vertical velocity seismogram of the RER station. This similarity suggests that they originated from a common physical process. The correlation coefficient between the VLP wave packets of CE1 and those of events E2 to E5 is higher than 0.73 and above 0.62 for the remaining events (Supplementary Table 1). Another similarity of these caldera collapse events is that all VLP wave packets showed very similar durations, of about 20 s (Fig. 2 and Supplementary Figs 1a and 2a,b). We furthermore observe a clear correlation between the maximum amplitudes of both VLP and ULP signals from E5 to CE48 (Supplementary Fig. 3b): the correlation coefficient is about 0.99 between VLP and ULP signals. It suggests that the sources of VLP and ULP signals are closely related. Finally, all these collapse events appeared to emanate from the same location. The horizontal location of Dolomieu Caldera is included in the fan-shaped area sandwiched between the white lines (Supplementary Fig. 5c) that indicate the polarization confidence limits. Considering the horizontal polarization measurements and their uncertainties (see Methods and Supplementary Table 2), the apparent back-azimuths of the E5-CE48 point toward both the Dolomieu Caldera and the fissure of the March eruption (Supplementary Fig. 5c) approximately 2 km southeast of the Dolomieu Caldera. Ground particle motions recorded for the CE1 from the two permanent GPS stations BONG and DERG (Supplementary Fig. 5c) indicate a source of deformation below the Dolomieu Caldera. Furthermore, C-CLVD earthquakes from April 1 to 5 at the Piton de la Fournaise volcano are observed below its summit^22^. Therefore, polarization measurements, GPS observations and the location of the epicenters all indicate that the likely source of the ULP signals is below the Dolomieu Caldera. If the source location of E1 and E2 may be slightly more eastward and may be associated with the magma lateral migration from the reservoir beneath the summit toward the eastern flank, we do not have constraint on the source location of E3 and E4 because polarization measurements were not possible due to low signal-to-noise ratio for these two events. However, the cross-correlation coefficient of the VLP signals of E2 and those of the CE1 is high (around 0.8) suggesting a similar source. The best constrained vertical polarization angle (*VPA*) from the collapse event E2 is 96.5 ± 6.3 suggesting a source located 0.1 km below the sea level ± 1.0 km (i.e, in a depth range between 1.1 km below the sea level and 0.9 km above the sea level). This depth range is fully compatible with the relocated seismicity of Massin *et al*.^22^ who showed that around 70% of the hypocenters are between the sea level and 0.8 km above the sea level from February 5 to May 1, 2007^22^. It is also consistent with depth range (∼0.5 ± 0.4 km above the sea level) of the first source of deformation located by the GPS analysis^25^ (Supplementary Fig. 6) and with the maximum depth associated with the low pressure (*P*_CO2_ + *P*_H2O_) range recorded in olivine melt inclusions (1.7 km below the summit)^33^. The similar location, shape and duration of the observed VLP signals recorded before and during the Dolomieu Caldera collapse indicates a repeating process, which clearly started before the surface subsidence.

### Moment tensor inversion

The CE2 event was greatly depleted in high-frequency energy content compared to standard earthquakes of similar magnitude (Supplementary Fig. 7), which can be explained by a source duration longer than that expected for common earthquakes of this magnitude^34^. The frequency content of all collapse events (CE1-CE48) suggests that the Dolomieu Caldera collapse involved several slow earthquakes with long source durations and slow ruptures.

**Figure 4:**
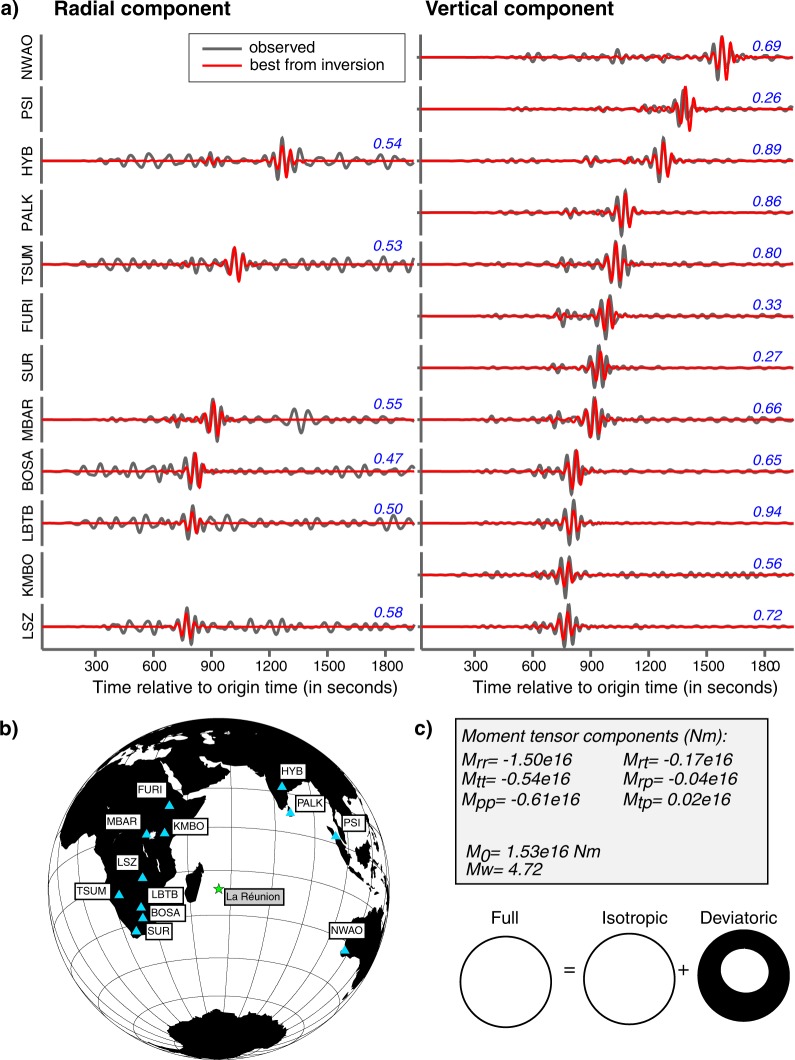
Results of the seismic moment tensor inversion. (**a**) Radial and vertical records in velocity filtered between 0.01 and 0.025 Hz (in grey) compared to the predicted seismograms (in red) obtained from the best inversion solution. The amplitudes are normalised by the maximum amplitude. The number shown in blue above each waveform is the correlation coefficient between observed and synthetic data. (**b**) Map of the location of the 12 seismic stations used in the inversion. (**c**) The optimum moment tensor components in the standard spherical coordinate system, the seismic moment, *M*_*0*_ and the moment magnitude, *M*_*W*_ are indicated. The focal mechanism from the best moment tensor is shown.

In order to constrain the mechanical processes occurring at depth during the caldera collapse, we performed moment tensor inversion (see Methods) from surface waves. We analysed the main collapse event (CE1) on April 5 and used horizontal and vertical seismograms (Fig. 4a and Supplementary Fig. 8a) from 12 teleseismic stations (Fig. 4b) band-pass filtered in the range 0.01–0.025 Hz. These long period data do not constrain the centroid location of the event, therefore we used our prior knowledge on the location of the caldera: longitude: 55.7119°, latitude: −21.2427°. We used the origin-time April 5, 2007 at 20:48:42, for the CE1 event. A grid search was performed over trial points at depths 5 to 15 with steps of 2 km. The best fit between the observed and the synthetic data is obtained for 5 km depth. A large range of source durations was tested for a triangle source time function. We plot the fit to the data against the half-duration of the triangle convolved with the data (Supplementary Fig. 8b). All source time functions with half-duration below 10 seconds show similar fit to the data. We observe a reduction in the fit towards higher half-durations, which only shows that the total duration of this event was not longer than 20 seconds. Interestingly, this duration is similar to the duration of the VLP wave packets on the seismic records (Fig. 2) and also consistent with slow earthquakes of long source durations (CE1-CE48) based on their frequency content. The centroid time delay is a parameter which can be used to estimate the source duration if the origin time and the source time function is well constrained^35^. In this study, the centroid time delay is −4 to −5 seconds (Supplementary Fig. 8c). It occurs 4 to 5 seconds before the origin time and around 5 to 6 s before the origin time from the bulletin of the volcanological observatory of Piton de la Fournaise (i.e. around 20:48:43). The origin time is usually calculated from the high-frequency *P*-waves. Therefore, results from the moment tensor inversion suggest a strong low frequency signal starting 5 to 6 seconds before the high frequency *P*-waves. However, it is difficult to estimate the source duration using solely the time shift of the optimum fit because the source time function is not very well determined and therefore large errors are possible^35^. For a 20 s duration, the moment tensor components and focal mechanism (Fig. 4c) show that the CE1 event was an earthquake with dominant isotropic volume decrease. The scalar seismic moment obtained from the moment tensor inversion is 1.53 × 10^16^ Nm using the Bowers and Hudson^36^ formula (see Methods) which corresponds to a *M*_*W*_ ∼ 4.7 using the equation 3.17 from Aki and Richards^37^. This value is in the range estimated from the surface-wave magnitude using the Main and Burton^38,39^ formula (see Methods) for earthquakes in the Aegean area: between 3.97 × 10^15^–7.35 × 10^17^ Nm. We used this relation between *M*_*S*_ and *M*_*0*_ to estimate the error bars on converting from *M*_*0*_ (or *M*_*W*_) to *M*_*S*_. We applied this formula^38^ to estimate the *M*_*S*_ value from the *M*_*0*_ of the CE1 event. We used the ± values in this formula to provide a crude estimate of uncertainty in *M*_*S*_. If we assume *M*_*0*_ = 1.53 × 10^16^ (which was obtained in moment tensor inversion), then *M*_*S*_ could be in the range between 3.5 and 5.3. This range is compatible with the *M*_*S*_ value of the CE1 event *M*_*S*_ ± 1*σ* ∼ 4.8 ± 0.2 directly determined from the amplitude of surface waves, which is an independent measurement.

The estimated moment tensor consists of a dominant isotropic component (57%) and a strong CLVD component (36%) characterized by a near vertical axis of compression and a small double couple (DC) component (7%). Supplementary Fig. 9 shows the results from the grid-search for time (relative to the origin) and depth obtained from the moment tensor inversion. The background is color-coded for the correlation between real and synthetic data. Each focal mechanism is colored based on the volumetric percentage. The highest correlations appear from −10 to 0 s (in dark purple) where the volumetric percentage is quite stable around 50–60% at all depths. At these low frequencies indeed, there is no depth sensitivity since between 5 and 15 km, similar mechanisms and correlations are observed. These results confirm the problem of resolving shallow depths from waveform data at low frequencies^40^. Therefore, to estimate the uncertainty of the isotropic, CLVD and DC components, we repeated the inversion using Jackknife method^41,42^ at the optimum depth (i.e. 5 km) as shallower depths are not resolved at these frequencies. We then plot the percentage of each component of the moment tensor against the number of stations used in the inversion (Supplementary Fig. 10). Using more than 10 stations, the moment tensor solution shows strong and stable isotropic and vertical CLVD components (Supplementary Fig. 10), which is in agreement with the low amplitude waveforms on the transverse components compared to the radial components (Supplementary Fig. 8a). This CE1 earthquake shows a large non-double-couple component. Such event can be characterised with the parameter *ε* defined by the following equation^18^: *ε* = −*λ*_*2*_/max(|*λ*_*1*_|, |*λ*_*3*_|) with *λ*_*1*_, *λ*_*2*_ and *λ*_*3*_ the eigenvalues of the deviatoric moment tensor and *λ*_*1*_ ≥ *λ*_*2*_ ≥ *λ*_*3*_. If *ε* = 0 it is a DC earthquake whereas for *ε* = −0.5 or *ε* = 0.5 it is a pure CLVD. The parameter *ε* is −0.42 in the case of the CE1 and the compression axis plunges more than 60°, which corresponds to a definition recently proposed for a vertical-CLVD earthquake^18^ with a dominant pressure axis. The parameter *k* defining the relative contribution of the isotropic component to the isotropic and deviatoric moment tensors^18^ is −0.63. Using the *k*-*ε* space (Fig. 6 of Shuler *et al*.^18^) the focal mechanism of the deviatoric fraction of the full moment tensor is close to the closing tensile crack end-member.

In summary, the CE1 earthquake shows a dominant non-double-couple component and both the isotropic and vertical CLVD components indicate a seismic source related to a near vertical pressure axis.

## Discussion

### Source mechanism: a ring-fault mechanism?

We can propose plausible source mechanisms to explain the combination of strong isotropic and vertical CLVD components based on our results and on published constraints from field geology, analogue and numerical modelling and seismology. The epicentral locations of earthquakes observed at a caldera can form roughly an elliptical annulus which is a characteristic of ring-fault seismicity^43^. Slip on curved normal faults can generate vertical-CLVD earthquakes with dominant pressure axes^44^. The existence of a ring-fault (i.e. curved or cone-shaped dip-slip fault) system can produce a vertical-CLVD earthquake^18^. Slip on ring-faults could also explain the anomalously long source duration time, a ring-fault seismicity, and observations of scarps of a ring-fault from the field.

The global average relationship between source duration *τ* and seismic moment *M*_*0*_ derived from body-wave waveform modelling for moderate-to-large shallow earthquakes is^45^ *τ* ≈ *4*.*52* × 10^−6^ *M*_*0*_^*1/3*^ where *τ* is expressed in s and *M*_*0*_ in Nm. A duration of approximately 1–4 s is predicted for the CE1 earthquake with *M*_*0*_ in the range 3.97 × 10^15^–7.35 × 10^17^ Nm from this equation. Therefore, the estimated duration of about 20 s for the CE1 earthquake during the Dolomieu Caldera collapse is clearly not common for an earthquake of such magnitude. Such a long duration for a *M*_*S*_ 4.8 seismic event supports a slow rupture on a ring-fault^18^. Interestingly, slow processes were also suggested during the 2000 Miyake-jima caldera collapse with source duration around 30–65 s^16,17^.

Several preserved polished surfaces were observed a few days after the first collapse on the scarps of a ring-fault indicating that the shallow subsidence was controlled by inward steep normal faults in sub-surface^46^.

The location of the seismicity is represented in Supplementary Fig. 5d. The epicentral locations of the earthquakes recorded at the Piton de la Fournaise volcano’s summit^22^ from February 5 to May 1, 2007 form roughly an elliptical annulus similar to that observed at the Rabaul caldera^43^ in Papua New Guinea during a seismic crisis period from late 1983 to mid-1985. The limits of the ring pattern may correspond to the caldera’s faults. The location of the epicenters observed at the Piton de la Fournaise volcano’s summit suggest an active ring-fault structure beneath the volcano’s summit between 0.8 km above the sea level and sea level.

We tested if slip on ring-fault structures can explain the incremental Piton de la Fournaise collapse. The theoretical scalar seismic moment can be described as^47^:

2 $${M}_{{0}}=\mu \,u\,S,$$

where *μ* is the rigidity, *u* is the average slip and *S* is the fault surface.

Slip on a ring-fault structure generates partial cancellation of moment release from different portions of the fault^44^. Assuming a ring-fault arc length of 360° and a dip of 85°, the composite scalar moment is 13% of that obtained by summing the scalar moments from each portion of the fault^44^. We also assume a fault surface approximated by a cylinder with a height of 1.7 km and radius of 0.2 km. The radius is derived from photographs obtained just after the CE1^5^ and from the extension of the seismicity at depth^22^. The height is estimated from the location of the shallowest C-CLVD earthquakes^22^. The cumulative seismic moment of the collapse events (CE1 to CE48) and of the events occurring before the caldera formation (E2 to E5) is around 2.28 × 10^16^–3.04 × 10^18^ Nm. Using Eq. (2) and assuming that *μ* = 30 GPa as Brooks *et al*.^48^ for slow earthquakes on Kīlauea volcano and as Ide *et al*.^49^ for various slow earthquakes, the required average slip *u* to explain the cumulative seismic moment is *u* = *M*_*0*_/(*μ* × *S* × 0.13) where *M*_*0*_ = 2.28 × 10^16^–3.04 × 10^18^ Nm, *μ* = 3 × 10^10^ Pa, and *S* ~ 2.1 × 10^6^ m^2^. Therefore, *u* is between 3 to 365 m. This estimated range of values is compatible with the observed final depth of the caldera (340 m)^27^ but the maximum value is lower than the estimated total collapse of 450 m computed by Michon *et al*.^50^ based on the final volume of the caldera divided by the observed surface of a piston. Due to the uncertainty on the dip angle, on the fault surface geometry and on the rigidity, this estimation above all shows that ring-fault structures can produce the incremental Piton de la Fournaise collapse. We applied the same approach to all the collapse events before and during the caldera formation. The results show a quasi-linear increase of the displacement from 16:19 on April 5 to April 7 (Fig. 3c) suggesting that the collapse already started at depth before CE1.

However, the dominant isotropic component obtained from the moment tensor inversion cannot be explained by a pure ring-fault mechanism. Therefore, one has to discuss other potential source mechanisms.

### Other types of potential source mechanisms

#### Mass-exchange mechanism

This mechanism, which could result from mass-exchange between specific types and orientations of magma reservoirs will produce a negligible volumetric component in the moment tensor^42^, so it is incompatible with the moment tensor inversion solutions.

#### Closing of tensile cracks

The relationship between the moment tensor and the volumetric change for opening or closing cracks, expansion or contraction of a spherical source is described by several authors^18,37,51^. We estimated the volume change associated with the model of a crack with the formula^51^: *M* = *ΔV*_*crack*_(*λ* + 2*μ*/3) where *M* is the isotropic part of the total seismic moment, *λ* and *μ* are the Lamé parameters and *ΔV*_*crack*_ is the volume change of the crack volume. In this equation, the isotropic component is considered to be 57% as for the CE1 event. This equation correspond to the case of a flat crack volume with arbitrary shape and curvature^51^. From this equation, we obtained *ΔV*_*crack*_ between 2.6 × 10^5^ and 3.5 × 10^7^ m^3^ using, and assuming *λ* = *μ*, *μ* = 30 GPa^38,48^ and using the isotropic part of the total seismic moment [2.28 × 10^16^–3.04 × 10^18^] × 57%~[1.30 × 10^16^–1.73 × 10^18^] Nm. This volume is up to around 39% of the 9 × 10^7^ m^3^ final volume of the caldera^27^. Therefore, this mechanism may contribute partly to the CE1 event.

#### Contraction of a spherical source

The isotropic volume change associated with all collapses can be estimated using the isotropic part of the estimated total seismic moment of all collapses (between 1.30 × 10^16^ and 1.73 × 10^18^ Nm), the following equation^51^: *M* = *ΔV*_*sphere*_(*λ* + 2*μ*) where *ΔV*_*sphere*_ is the volume change of the spherical reservoir, and assuming *λ* = *μ* and *μ* = 30 GPa. The total isotropic volume change of the reservoir associated with all collapses is between 1.4 × 10^5^ and 1.9 × 10^7^ m^3^, which is up to around 21% of the final caldera volume. Although, this mechanism may contribute partly to the CE1 earthquake, its potential contribution is around 54% of the closing tensile-crack mechanism.

#### Piston-like model

At Miyake-jima, the source of the VLP signals prior to and after the first caldera collapse was attributed to the pressurisation of a magmatic reservoir impacted by a falling rock column^4,13^. In this model, the downward displacement of the piston provokes the over pressurisation of the magma storage volume and generates VLP signals. A piston model was also used for the 2014–2015 Bárðarbunga Caldera collapse. In this study, both the dominant isotropic component (57%) and the large CLVD component (36%) of the CE1 event indicates a downward contraction which could be due to the near vertical compression of the magma reservoir. A collapsing piston model may therefore explain this near vertical compression and is also compatible with a crack like geometry^4^ and with previous results from the analysis of the seismicity and of the GPS data.

Earthquakes with C-CLVD and normal fault mechanisms occurred just before the main collapse event (CE1) from April 1^st^ to 5^th^ between the sea level and 0.8 km above the sea level^22^. Massin *et al*.^22^ considered the C-CLVD earthquakes as a precursor of the Dolomieu Caldera collapse. GPS data from the geodetic permanent network were processed by Peltier *et al*.^25^ using 3D elastic model based on the mixed boundary element method^52^ combined with the neighbourhood algorithm^53^. A migration of the source of deformation was observed from February 21^st^-March 29^th^ 2007 to April 5^th^–6^th^ (Supplementary Fig. 6). The pre-eruptive March inflation was explained by an ellipsoidal source of pressure located beneath the Dolomieu summit at ~0.5 ± 0.4 km above the sea level, whereas the source of deflation was located beneath the Dolomieu summit at ~2.3 ± 0.4 km on April 5^th^–6^th^. Interestingly, recent results from Holohan *et al*.^12^ using discontinuum mechanics models applied to the March-April 2007 eruption of Piton de la Fournaise show that host-rock fracturing (i.e. non-elastic deformation) during magma withdrawal from the magma reservoir can explain the migration and change of shape determined with an elastic-deformation source. They proposed that deflation associated with host-rock fracturing can be the cause of the underestimation of the true magma reservoir depth on April 5^th^–6^th^. Holohan *et al*.^12^ proposed that the rock column above the magma reservoir experienced an initial phase of sub-surface fracturing from April 2^nd^–5^th^, then from April 5^th^–6^th^ a piston-like collapse occurred through a ring-fault system.

Slip on ring-faults could explain some observations such as anomalously slow source processes, observations from the field and the characteristic ring-fault seismicity. However, the dominant isotropic component obtained from the moment tensor inversion cannot be explained by a pure ring-fault mechanism whereas it could be explained by a piston-like model. Based on the previous arguments, our preferred model to explain the 2007 Piton de la Fournaise Caldera collapse is a piston-like collapse occurring through a ring-fault system. In this model, a rock column overlying a magma reservoir is delimited by ring-faults and collapsed due to the decrease of pressure of the magma reservoir associated with the lateral magma migration. This model is fully compatible with recent numerical modelling^12^ suggesting a possible piston-like collapse on April 5^th^–6^th^, 2007 during Piton de la Fournaise collapse once the through-going ring-fault system has developed.

### Origin of VLP and ULP seismic signals

**Figure 5:**
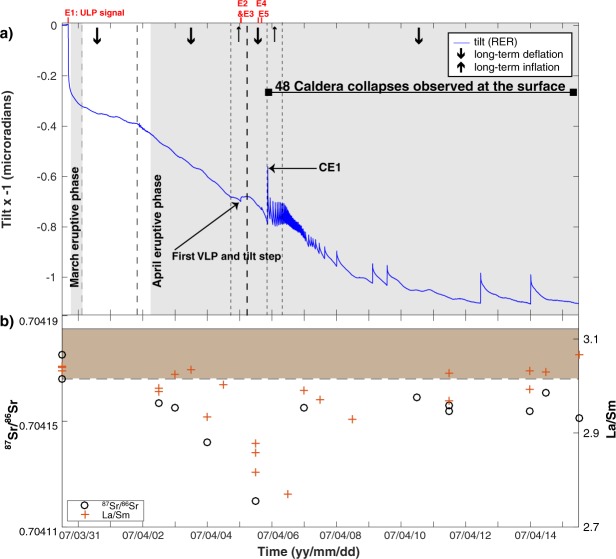
Comparison of the tilt signal long-term variation and the geochemical time series. (**a**) Tilt determined from the seismic station RER^24^. The blue trace is the tilt signal corrected for tide effects. (**b**) *Sr*^87^*/Sr*^86^ and *La/Sm* isotope ratios^33,57^. The brown rectangle shows the range of *Sr*^87^*/Sr*^86^ in bulk Piton de la Fournaise lavas between 2001 and 2006^57^.

The tilt analysis at the RER broadband seismic station was performed in a previous study and details about the method is fully described in this study^24^. Tilt data^24^ permits the identification of several key seismic events that preceded the April 5 main caldera collapse (Fig. 5a and Supplementary Fig. 4c). Event E1 (Supplementary Fig. 1b) recorded by the RER seismic station occurred on March 30, i.e., five minutes after the onset of the pre-eruptive seismic crisis that led to the March 30 eruption^27^, and was followed by a long-term change in tilt. While the seismic record from the RER station shows a long-term edifice deflation from March 30 at 16:31 to April 1 at 19:52, the GPS station FERG (see its location on Supplementary Fig. 6) measured an eastward displacement^54^. This eastward displacement starts at 16:30 at FERG and is interpreted as the result of slip of the Eastern Flank of Piton de la Fournaise^54,55^. The long-term change in tilt variation may be a result of a rapid dyke injection^55^ beneath the summit cone followed by its lateral migration toward the eastern flank. Tilt analysis of the RER station reveals a stronger long-term edifice deflation between April 1 at 19:52 and the beginning of the eruptive phase on April 2 (Figs 3 and 5a of Fontaine *et al*.^24^). This deflation may be induced by magma lateral migration toward the April eruptive vent. The tilt signal related to the lateral magma migration is only characterized by a period of deflation whereas shorter tilt signals associated with the collapses events show inflation-deflation cycles (Fig. 5a). Furthermore, this tilt signal is not accompanied by VLP seismic signals as observed during the collapse events. The deflation continued until April 5 when the E2 event corresponding to the first tilt-step change (Fig. 5a) accompanied by VLP seismic signals (Fig. 5a and Supplementary Fig. 4c) is observed. Events E2 to E5 (Fig. 5a and Supplementary Fig. 1b) started around 20 hours before the collapse onset at the surface and can be interpreted as inflation-deflation cycles (see Fig. 3b of Fontaine *et al*.^24^), similar to those observed during the following collapse events (CE1 to CE48) (Figs 3d and 5a). Each of the E2–E5 events correspond to both a VLP wave packet of around 20 s duration and a tilt signal whereas E1 is a tilt-only signal. These four events (E2 to E5) are considered as collapses at depth prior to the collapse onset at the surface based on the evidence suggested from the analysis of the waveform, duration and location similarity. The first precursory collapse at depth (E2) occurs immediately after a long-term period of edifice deflation with a strong decrease of around 0.7 μrad from March 30 at 16:35 to the time of the E2 event^24^. Therefore, we interpret the cause of the caldera collapse episode as due to a pressure decrease in the magma reservoir.

A fast increase of the magma outflow rate occurs between April 5 at 10:00 with 75 m^3^/s from MODIS data^56^ and April 6 at 12:00 with a value higher than 200 m^3^/s from a field survey^27^. From April 8 to May 1 the eruption continued at a low intensity^50^. The time difference *∆t* between two successive tilt signals related to collapse events was measured in a previous study^50^. *∆t* clearly decreased, whereas the magma outflow rate increased from April 5 20:48 to April 6 at 12:00. *∆t* then increased, whereas the magma outflow rate decreased until April 8 at 00:09. Our study (Fig. 5a and Supplementary Fig. S4) suggests that the increase of *∆t* occurs during a longer period: until April 12 at 11:05. Interestingly, an increase of the magma outflow rate was then determined from MODIS data^56^ with 30 m^3^/s on April 11 at 18:45 to 55 m^3^/s on April 12 at 10:04 which may explain a decrease of *∆t* between CE47 and CE48. The amplitude of the tilt signal decreases from April 5 at 20:48 until April 6 at 12:00, whereas the magma outflow rate increases during this period. Then, the amplitude of the tilt signal increases from April 6 at 12:00 until April 12 at 11:05, whereas the magma outflow rate decreases during this period. Our study confirms a clear link between the eruptive activity and the occurrences of the tilt signals. Such relationship between *∆t* and the magma outflow rate in which *∆t* is inversely proportional to the magma outflow rate, is in agreement with a piston-like model^4^.

A previous study^24^ reported that the volcanic edifice experienced 2 periods of slight inflation according to tilt data: between April 4 at 17:00 and April 5 at 7:00 and between April 5 at 20:48 and April 6 at 8:00. These slight inflation periods, in spite of the large ongoing magma withdrawal, can arise from a sudden and short-lived deep magma input^33^ which could cause a sudden pressure increase of the magma reservoir. A major shift of the isotopic (*Sr*^87^*/Sr*^86^) and incompatible trace element ratios (*La/Sm*) is observed in lavas^33,57^ erupted during the period April 4 to 6 with respect to those erupted during the whole 1999-March 2007 period (Fig. 5b). This downward shift in the ratio of *Sr*^87^*/Sr*^86^ and *La/Sm* during the period of caldera collapse suggests a new injection of deep magma into the shallow (above sea level) part of the plumbing system^33^.

Precursory collapses at depth were only reported before the caldera formation of Miyake-jima and in this study we show their occurrence before the 2007 Dolomieu Caldera formation. What could explain their occurrence at both of these volcanoes and not prior to other caldera formation such as the 2014–2015 Bárðarbunga caldera development? As mentioned earlier, multiple analogue experiment of caldera formation from Roche *et al*.^8^ highlight the importance of the roof aspect ratio (i.e. roof depth/piston diameter) in the fault formation and in the onset of the caldera formation. For low aspect ratio (≤1), the caldera collapsed as a coherent piston and the caldera surface subsidence is observed from the onset of the caldera formation. This was likely the case during the Fernandina caldera formation in 1968^31^ where the reservoir diameter was around 3.2 km (ref.^23^) and the depth of the magma reservoir roof was estimated to be shallower around 1 km corresponding to a roof aspect ratio of around 0.3. For the 2014–2015 caldera development of Bárðarbunga, the depth of the magma reservoir was estimated to be much deeper (~12 km)^6^. This depth was constrained from lava chemistry, surface gas composition and geodetic modeling, providing a roof aspect ratio of 1.6 considering the diameter was around 7.5 km (ref.^6^). In the case of the 2007 Piton de la Fournaise collapse, we obtained a higher aspect ratio around 4.25. Interestingly, the roof aspect ratio was also high (between 1.9 and 3.8) for the 2000 caldera formation at Miyake-jima^23^. This therefore suggests that precursory collapses at depth at Piton de la Fournaise and at Miyake-jima are likely due to the roof aspect ratio.

Although the 2018 Kīlauea Caldera collapse episode was not reported as a piston-like collapse^7,58^, the caldera floor subsidence was likely induced by the lateral withdrawal of magma from the summit reservoir system^7^ as proposed in this study. Similarly to for the Dolomieu Caldera collapse, the collapse episode was characterized by several collapse events and moment tensor inversions suggest a complex source mechanism associated with dominant changes in volume^7^.

### Caldera collapse processes at Piton de la Fournaise

**Figure 6:**
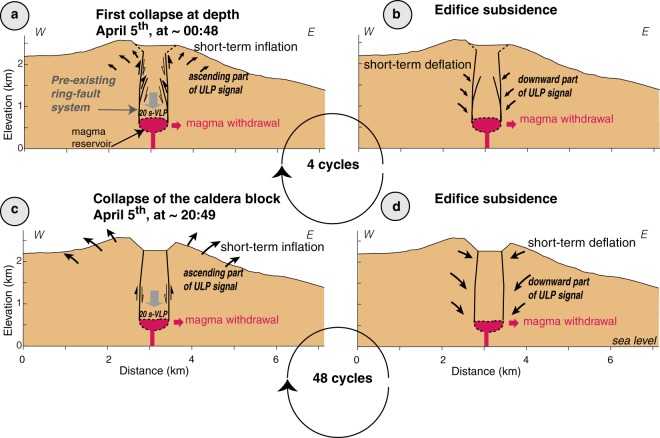
A conceptual model proposed to explain the occurrence of both VLP and ULP signals. (**a**) Collapse at depth. VLP and ascending ULP signals are generated. (**b**) Edifice subsidence and relaxation producing the last part of the ULP signal. (**c**) Collapse of the caldera block generates VLP signals and ascending ULP signal. (**d**) Edifice subsidence and relaxation inducing the last part of the ULP signal.

From the observations presented above, our preferred model to explain the caldera formation is illustrated in Fig. 6. Lateral magma migration towards the distant April eruption site and subsequent onset of a reservoir or a network of dykes and sills^59^ collapse at depth were possibly triggered by the pressure decrease in the magma reservoir. Collapse at depth occurred following four cycles (E2 to E5) each characterised by two steps. Firstly, a downward motion of a rock column without visible surface rupture through a ring-fault system surrounding a magma reservoir that generated the first part of an ULP signal: a tilt-step signal accompanied by VLP signals (Fig. 6a). The first part of the ULP signal is likely due to the response of the volcanic edifice to the downward motion of the rock column. Secondly, the slip stopped on the ring-fault, and the subsidence of both the caldera block and the edifice generated the last part of the ULP signal (Fig. 6b), which could be related to stress relaxation within the volcanic edifice. After this initial phase at depth, the caldera collapse occurred in 48 repeating cycles (CE1 to CE48) generating both VLP and ULP signals similar to those of the precursory phase (Fig. 6c,d) but accompanied by surface rupture. Waveforms, spectral content and source location of the seismic signals related to the caldera collapse events show homogeneous patterns, suggesting a similar and repeating volcano-tectonic process for the formation of the VLP signals. The strikingly constant duration of the VLP wave packets (around 20 s) related to the collapse events and their occurrence before the collapse initiation suggest a physical control of the volcanic edifice. The source mechanism from the main collapse, observations of slow source processes, observations from the field and the characteristic ring-fault seismicity indicate that the collapse could result from a piston-like downward motion occurring through a ring-fault structure surrounding a magma reservoir.

Analysis of ULP signals accompanied by VLP signals recorded by a nearby broadband seismic station can thus provide important constraints on the timing of the 2007 Piton de la Fournaise Caldera collapse onset. A total of 44 collapse events was reported before this study^50^ from the analysis of the seismic records of RER station between April 5 at 20:48 to April 8 at 00:09. In this study, we analysed the seismic records of RER during a longer time span and detected 4 precursory collapse events (E2–E5) occurring at depth before the main event and 4 other events occurring at the end of the sequence (CE45–CE48) after April 8 at 00:09. This study demonstrates the possibility of detecting the first collapse (E2 event) at depth approximately 20 hours before the surface rupture and the caldera formation from the identification of ULP signals accompanied by VLP signals. Furthermore, the likely source mechanism of the caldera collapse can be identified from both records from this local very sensitive seismic station and teleseismic stations from the global seismic network. These observations are crucial for volcanic hazard assessment and particularly for volcano early-warning systems that may help in the decision of visitors and/or population evacuation at critical volcanoes. This study shows the importance of considering at least a broadband seismic station in volcano monitoring, particularly for those that can be potentially devastating by their eruptions or collapses.

## Methods

### Surface-wave magnitude and scalar seismic moment

*M*_*S*_ was determined from the following classic equation^37^:

3 $${M}_{S}={\mathrm{log}}_{10}(A/T)+1.66\,{\mathrm{log}}_{10}\,{\Delta }+3.3,$$

where *A* is the ground amplitude of Rayleigh wave in the vertical component in microns; *T* is the period near 20 s; and *Δ* is the epicentral distance in degrees.

Bowers and Hudson^36^ proposed to define the total scalar seismic moment as *M*_*tot*_ = *M*_*ISO*_ + *M*_*DEV*_ where *M*_*ISO*_ = |(*m*_*1*_ + *m*_*2*_ + *m*_*3*_)/3| is the isotropic moment tensor and *M*_*DEV*_ = max(|*m*_*j*_ − (*m*_*1*_ + *m*_*2*_ + *m*_*3*_)/3|; *j* = 1, 2, 3) is the deviatoric moment tensor, and *m*_*1*_, *m*_*2*_, and *m*_*3*_ are the eigenvalues of the seismic moment tensor with *m*_*1*_ ≥ *m*_*3*_ ≥ *m*_*2*_.

We estimated the scalar seismic moment of each collapse event (CE1 to CE48) and of the events occurring prior to the caldera formation (E2 to E5) from the calculated *M*_*S*_ values using the following formula^38,39^:

4 $${\mathrm{log}}_{10}{M}_{{0}}=(1.206\pm 0.105)Ms+(10.970\pm 0.632)$$

### Moment tensor inversion

To better constrain the seismic source associated with the main collapse event, we analysed three-component broadband data from permanent stations retrieved from the Incorporated Research Institutions for Seismology Data Management Center (IRIS DMC). Seismic stations with epicentral distances up to 60 degrees from the Dolomieu Caldera were selected. From an initial set of 50 stations only ones with signal–to–noise ratio (from the power spectrum) greater than 2 were considered. Subsequently, the quality of all waveforms was checked manually. The time window length is restricted to 2000 seconds from the origin time, which covers the full waveform for all seismic stations. We used a source time function with a triangle shape of 10–second half–duration, i.e. total duration of 20 seconds as an anomalously long source duration was suggested by the low frequency content of the CE1 event and as a grid-search for source time function duration provided an upper bound for the source duration of 20 s (Supplementary Fig. 8b).

The method we adopt for moment tensor inversion can be described as follows: six elementary seismograms^60^ have been calculated on a set of grid nodes below the Dolomieu Caldera. Each elementary seismogram represents a basic mechanism. A linear combination of these mechanisms can create any arbitrary moment tensor. We ran a grid search^61,62^ over trial points beneath the caldera to find the optimum depth. These points start at depth 5 km and end at depth 15 km with steps of 2 km. The optimum time is searched by shifting the seismograms around the hypocenter time. We did not search for the lateral location of the event because we know the location of the caldera. We used the PREM^63^ velocity model and the Mineos package^64^ to generate synthetics data. The optimum depth is at 5 km, the shallowest grid point. The shallower depths were not searched since the vertical dip-slip components of the moment tensor cannot be resolved at long periods. The combination of the grid–point and time–shift that provides the highest correlation between real and synthetic data is considered as optimum centroid depth and time. The best solution from the full moment tensor inversion is represented in Fig. 4c. To estimate the uncertainty of the moment tensor components we repeated the inversion using Jackknife method at the optimum depth (Supplementary Fig. 10).

### Polarisation analysis of particle motions

We performed a polarisation analysis using the three-component seismic station RER. Repeating collapse events recorded at RER were characterised by notable ultra-long period signals (Supplementary Figs 3 and 4b,d) with frequencies in the range 0.003–0.01 Hz. They exhibited clear polarisations in both horizontal and vertical planes. The waveforms were band-pass filtered between 0.003 and 0.01 Hz because the tilt effect is observed to be dominant at frequencies lower than the lower corner frequency of the RER seismometer: below 1/360 Hz^24^ and to minimise the effects of local stratigraphy, as well as the local volcano topography that generally affects the higher frequencies^65,66^ and the swell-induced micro-seismic noise^67^. Considering the very low to ultra-low frequency range used, the particle motion points approximately toward the source^68^. This frequency band was also chosen because the high particle motion linearity suggests a *P*-wave nature of the signal rather than a combination of surface and scattered waves. We therefore focused the polarisation analysis on the ULP signals (i.e. 0.003–0.01 Hz). The polarisation analysis was performed using a 667 s window around the onset of the signal. The covariance matrix resulting from this analysis was determined from the principal component analysis (PCA) applied to the three components seismic records^67,69^ (Supplementary Information). The horizontal and vertical polarisation angles were measured from PCA of the particle motion (Fig. S8a,b). Uncertainties of both the apparent horizontal polarization angle (*E*_*BAZ*_) and the vertical polarization angle (*E*_*VPA*_) were computed as described in the supplement text material following a formula proposed by D. Reymond^69,70^.

Two other effects: the sensor misorientation and the presence of seismic anisotropy in the volcanic edifice could also disturb the signal polarisation. At RER permanent seismic station, the exact azimuths of the so-called N-S and E-W components have been checked with a gyrocompass and found to be N2.3° and N89.5°, respectively. This small sensor misorientation of each STS-1 horizontal component was neglected during the analysis. Power spectral densities computed at RER station show that the micro-seismic noise related to the swell activity occurred at higher frequencies than the frequencies considered during the polarisation analysis: mostly between 0.05 and 0.33 Hz (ref.^71^). The presence of seismic anisotropy in the volcanic edifice could also result in a deviation of horizontal polarization angle^69^. However, the horizontal polarization angles of the E5-CE48 point toward the fissure of the March eruption (Supplementary Fig. 5c). Therefore, in this study, we suppose that the anisotropic contribution is negligible on the horizontal polarization angles.

The Supplementary Table 2 provides the results of the polarisation analysis for all events (E1 to CE48) except E3 and E4. E3 and E4 were not considered due to signal–to–noise ratio <2.

## Supplementary information

Supplementary Information

## Data Availability

The seismological data used are available from GEOSCOPE: 10.18715/GEOSCOPE.G, IRIS/IDA: 10.7914/SN/II, the Global Seismograph Network: 10.7914/SN/IU, the New China Digital Seismograph Network: 10.7914/SN/IC and the Global Telemetered Seismograph Network: 10.7914/SN/GT. The GPS data is available from the volcanological observatory of Piton de la Fournaise (http://volobsis.ipgp.fr).
