# Supplementary material for: Very- and ultra-long-period seismic signals prior to and during caldera formation on La Réunion Island
Source: Sci Rep. 2019 May 30;9:8068. doi: 10.1038/s41598-019-44439-1 (PMC6543087; doi:10.1038/s41598-019-44439-1)
Supplement: Supplementary file 1 — Supplementary Information [file 41598_2019_44439_MOESM1_ESM.pdf]

Supplementary information to the paper:

Very- and ultra-long-period seismic signals prior to and during caldera formation on La Réunion Island

F. R. Fontaine[1,2]*, G. Roult[1], B. Hejrani[3], L. Michon[1,2], V. Ferrazzini[1,4], G. Barruol[1,2], H. Tkalčić[3], A. Di Muro[1,4], A. Peltier[1,4], D. Reymond[5], T. Staudacher[1,4] and F. Massin[6]

[1]Université de Paris, Institut de physique du globe de Paris, CNRS, F-75005 Paris, France.

[2]Université de La Réunion, Laboratoire GéoSciences Réunion, F-97744 Saint Denis, France.

[3]Research School of Earth Sciences, The Australian National University, Canberra, ACT 2601, Australia.

[4]Observatoire volcanologique du Piton de la Fournaise, Institut de physique du globe de Paris, F-97418 La Plaine des Cafres, France.

[5]CEA/DASE/Laboratoire de Géophysique, Commissariat à l'Energie Atomique, BP 640 98713 Papeete, Tahiti, French Polynesia.

[6]Swiss Seismological Service, ETH Zurich, Sonneggstrasse 5, CH-8092, Zurich, Switzerland.

Supplementary Figures

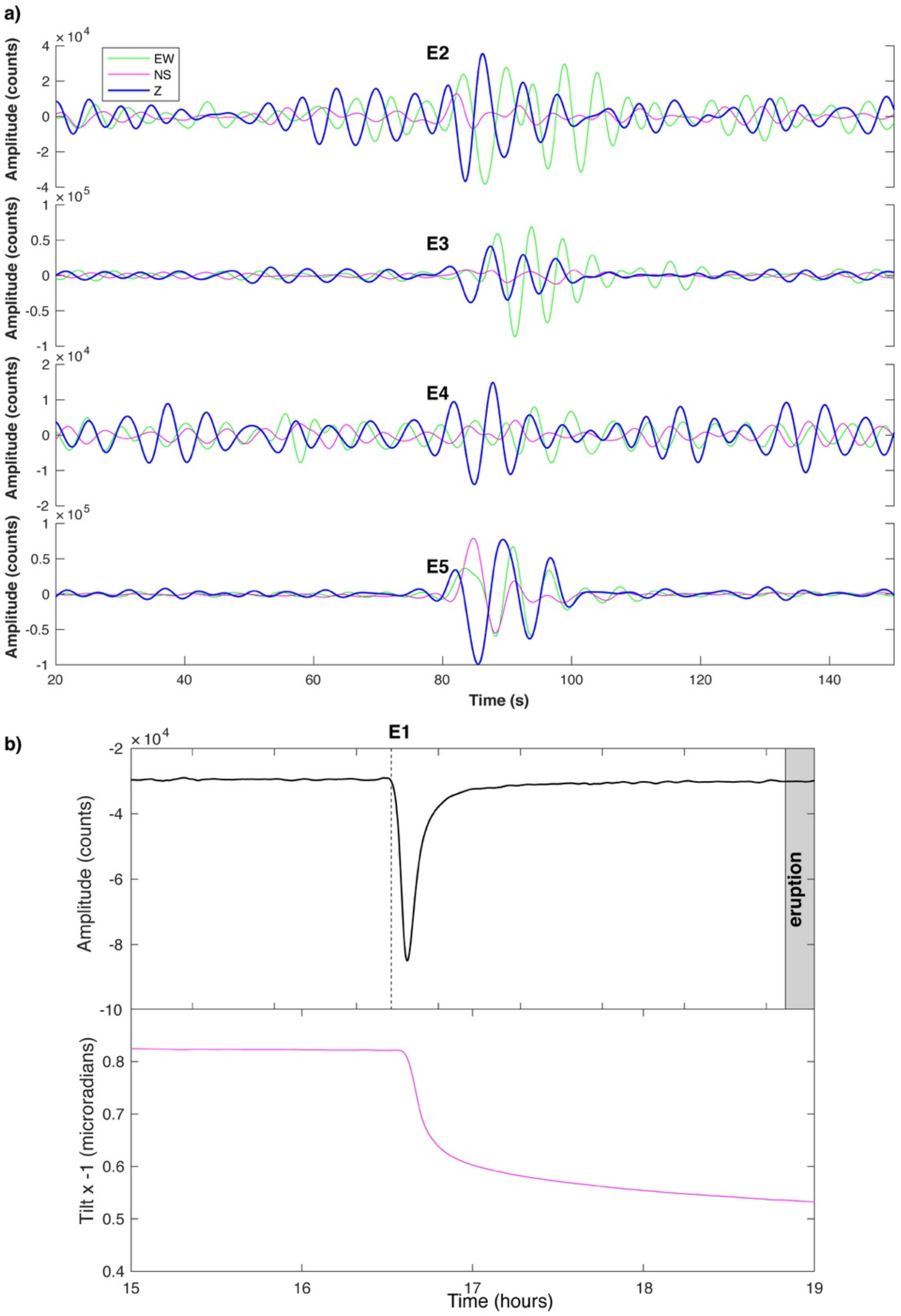

Figure S1. Seismic signals recorded at RER station before the Dolomieu Caldera collapse. (**a**) Blow-up of VLP signals for E2, E3, E4 and E5. Recordings of the three broadband raw components (Z vertical, North-South and East-West) after applying a 5% Hanning taper, removal of the mean, removal of the trend, and band-pass filtering between 0.005 and 0.20

with a zero-phase 4-pole Butterworth filter. (**b**) ULP seismic signal associated with the E1 event observed at the RER station. The plot at the top show the N-S seismic records (data in counts) for the very-long-period (VH) channel (i.e. sampling rate of 0.1 Hz). The figure at the bottom represents the N-S tilt signal computed at RER by Fontaine *et al.*[24] using a similar procedure adopted by Genco and Ripepe[73]. The records starts on March 30, 2007 at 15:00. The vertical dashed line indicates the onset of the E1 event.

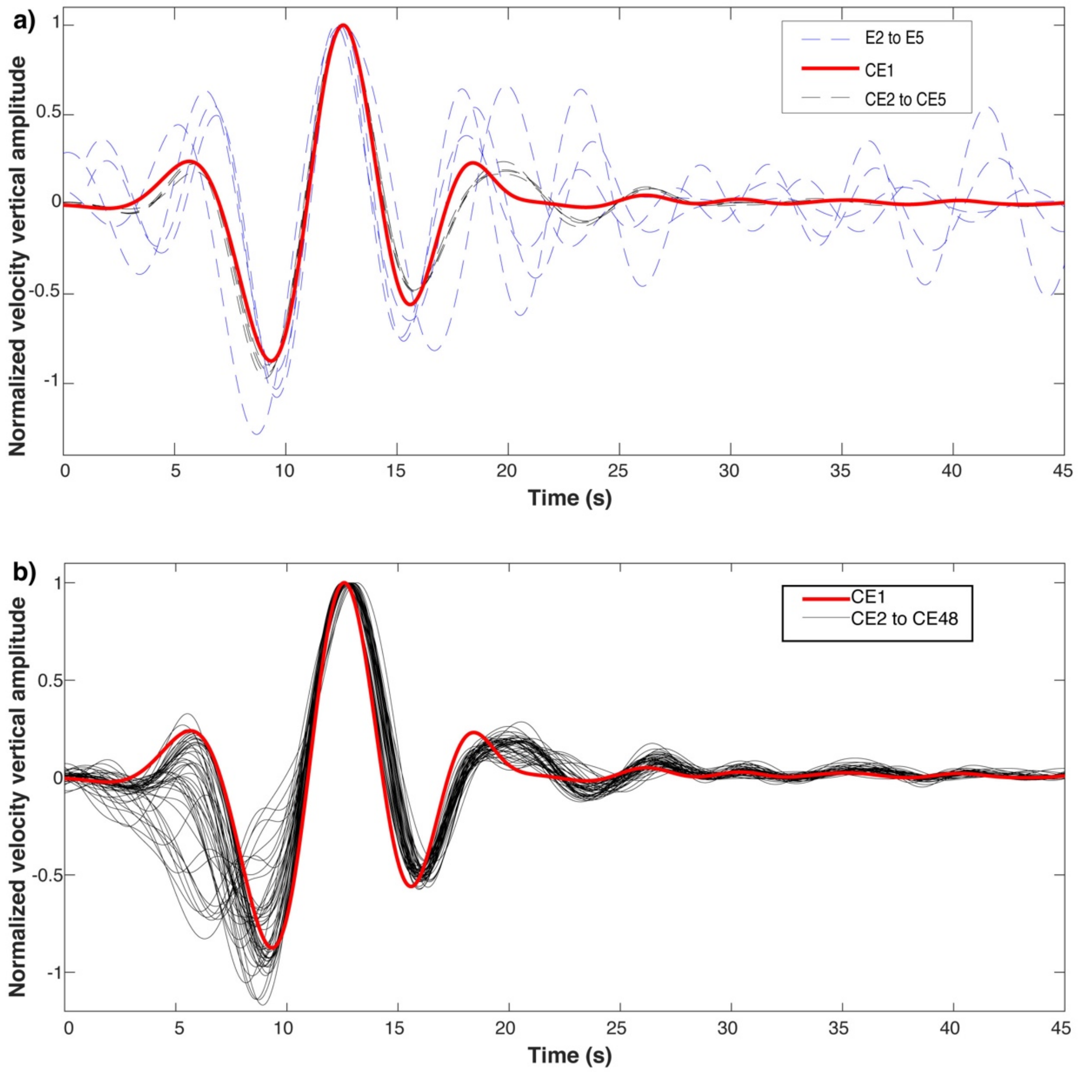

Figure S2. Similarity of VLP wave packets. (**a**) VLP wave packets observed from E2 to CE5. Seismograms from the vertical component of the RER station after applying a 5% Hanning taper, removal of the mean, removal of the trend, and band-pass filtering between 0.005 and 0.20 with a zero-phase 4-pole Butterworth filter. The instrumental response was not removed. VLP signals from the CE1 (in red) is compared to those occurring before this event: from E2 to E5 (in blue) and VLP signals observed after this main event: from CE2 to CE5 (in black). (**b**) VLP signals occurring from CE1 to CE48. The signal was processed similarly than in (a). The solid red line shows the VLP signals from the CE1 whereas the solid black lines represent the VLP signals observed from CE2 to CE48.

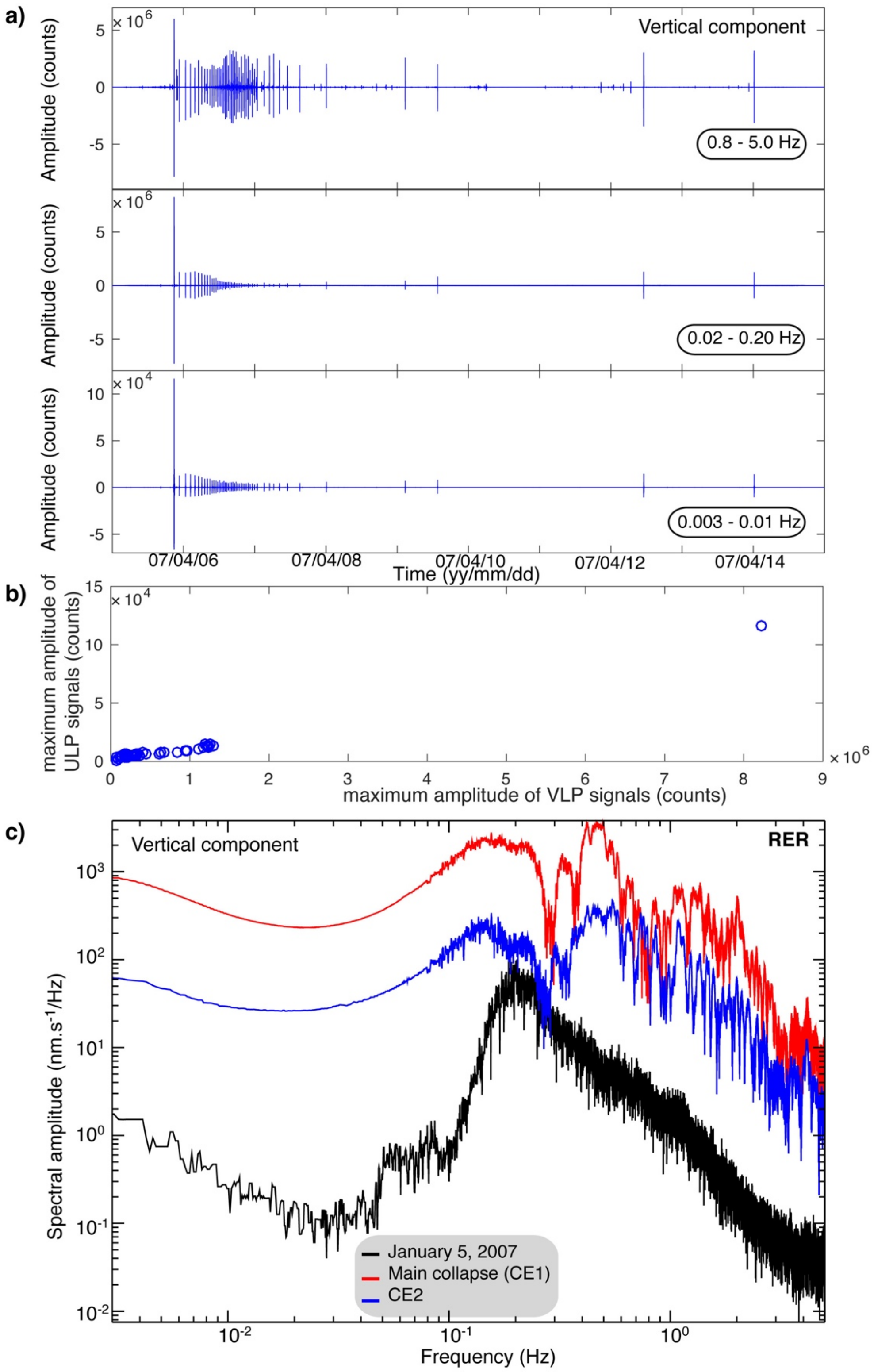

Figure S3. Seismic signals recorded at RER station during the Dolomieu Caldera collapse. (**a**) Waveforms band-pass filtered in three different frequency ranges: i) between 0.8 and 5.0 Hz to see the presence of short period signals (SP), ii) between 0.02 and 0.20 Hz to identify the VLP signals and iii) between 0.003 and 0.01 Hz to highlight the ULP signals (ULP). (**b**)

The maximum amplitude of VLP signals is shown as a function of the maximum amplitude of ULP signals from E5 to CE48. (**c**) Amplitude spectra of the vertical record computed using a 6000-s time window for different periods: before the collapse and without eruption (on January 5, 2007), during the main collapse (CE1) and during the CE2.

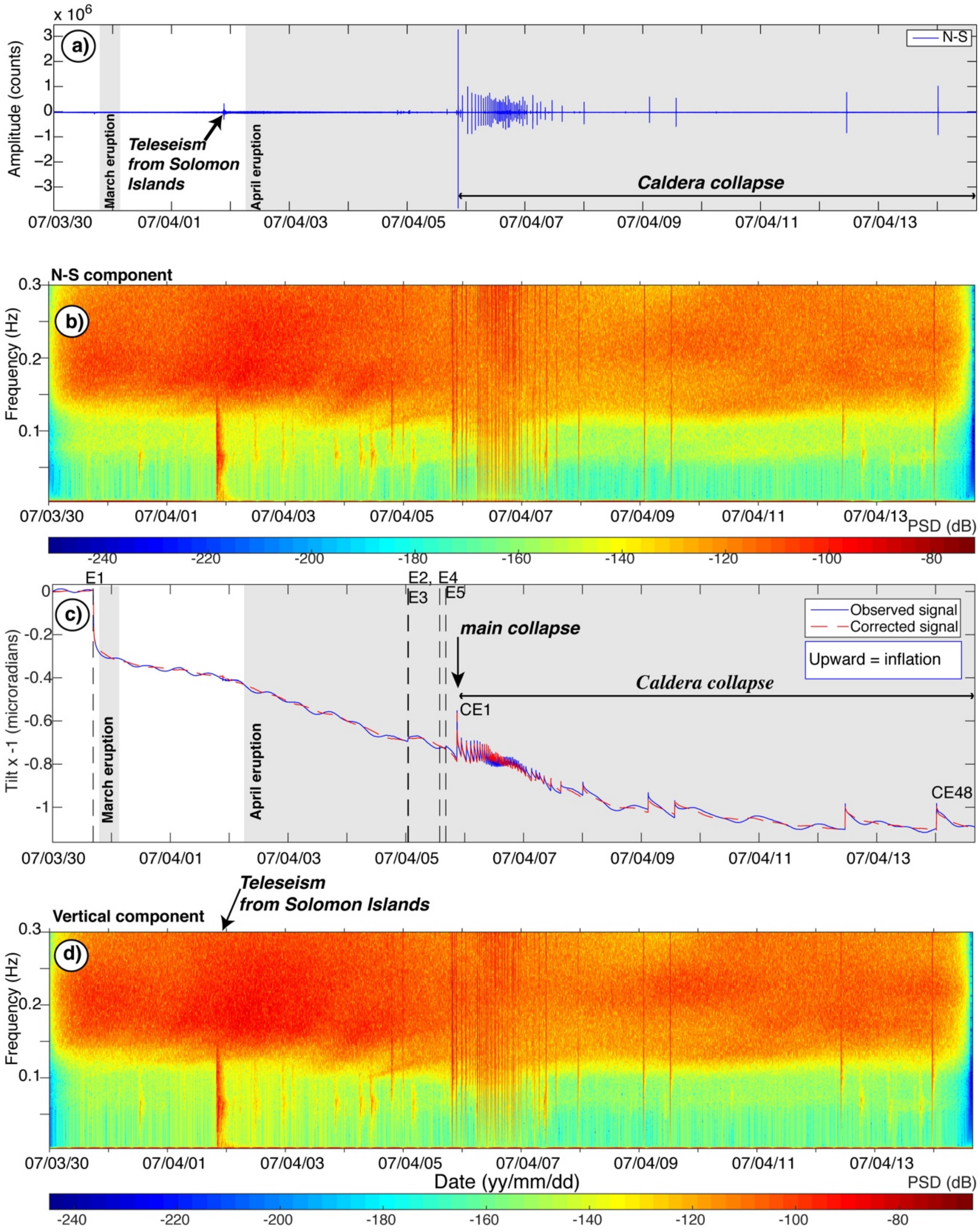

Figure S4. VLP and ULP seismic signals observed at the RER station. (**a**) Recording of the north-south component (data in counts) for the long-period (LH) channel (i.e. sampling rate of 1 Hz) of RER station from March 30. Grey boxes show the period of eruptions. (**b**) Spectrogram of N-S component showing the occurrence of VLP and ULP signals with higher amplitude than the micro-seismic noise. The signal was divided in sections of 512 points with 25% overlap of the sections, and sampling frequency of 1 Hz. The power spectral density

(PSD) is represented in units of dB related to 1 (m s^{-2})^2 Hz^{-1}. (**c**) Observed (in blue) and predicted (in red) tilt determined from the north-south component of RER station[24]. Upward: inflation and downward: deflation. (**d**) Spectrogram of the vertical record showing the occurrence of VLP and ULP signals both before (E1 to E5) and during the collapse episode (CE1 to CE48). The signal was divided similarly than in (b) and the PSD is represented in the same unit than in (b). Grey boxes show the period of eruptions.

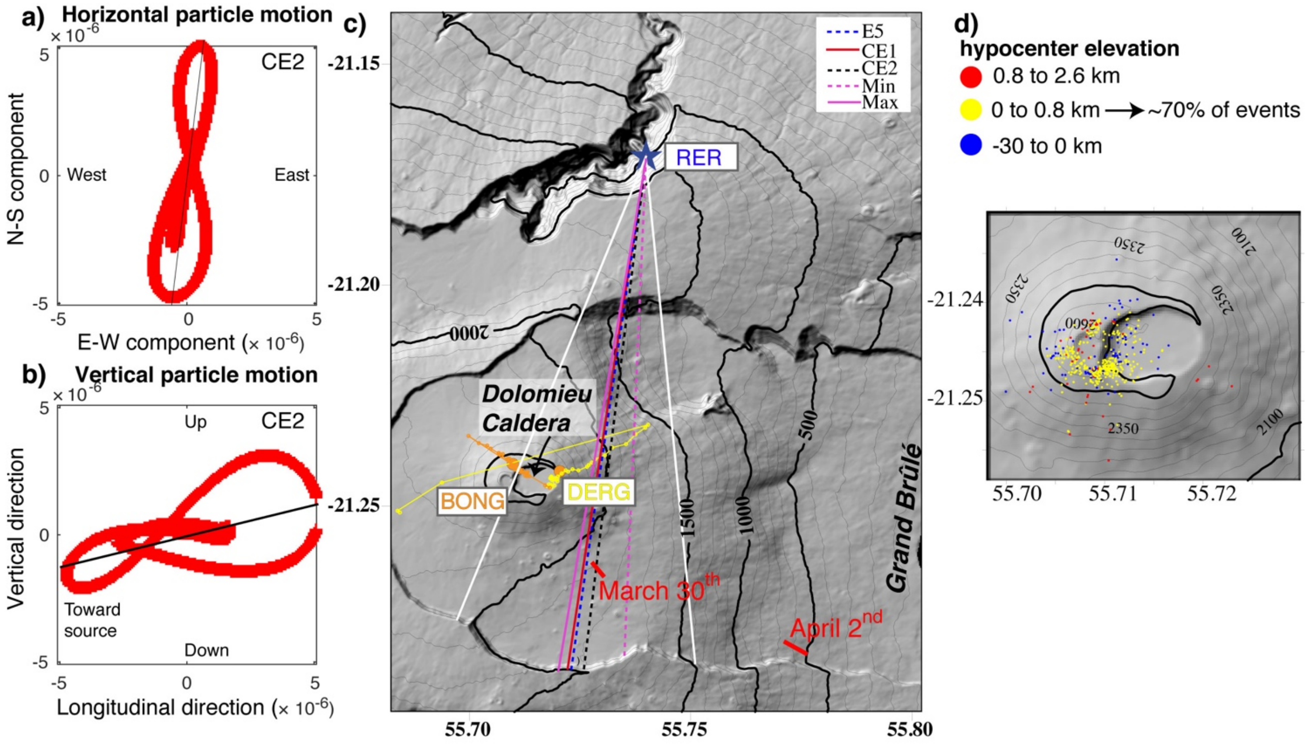

Figure S5. Polarisation directions of ULP signals and relocated seismicity. Horizontal (**a**) and vertical (**b**) particle motions showing the observed polarization for the CE2 event at the GEOSCOPE station RER. The solid black line shows the estimated polarisation direction. The horizontal polarisation angle is 186.9 ± 8.9 whereas the vertical polarization angle is 103.7 ± 18.5. (**c**) Measured back-azimuths at the RER seismic station. Dashed and solid lines in pink show the minimum and maximum back-azimuth measured from E1 to CE48. White lines indicate the polarization confidence limits considering the uncertainties of all the measurements of back-azimuth (Supplementary Table 2). Measured back-azimuths of the CE1 and the CE2 are respectively shown with a solid red and a dashed black line, whereas for the E5 it is represented with a dashed blue line. Ground particle motion recorded for the CE1 from RER and from BONG and DERG permanent GPS stations are also indicated in orange and yellow respectively. The time period corresponding to the data from these GPS stations are starting from April 5, 2007 at 20:48 and the time window length is 1000 s. Fissures corresponding to the March and April eruptions are located. (**d**) Relocated seismicity from

February 5 to May 1, 2007[22]. Hypocentre depth beneath the surface is relative to the sea level. Maps of Piton de la Fournaise was generated with the SURFER v10 software (http://www.goldensoftware.com/products/surfer) and the digital elevation model of the IGN (http://professionnels.ign.fr/bdalti, 25-m resolution).

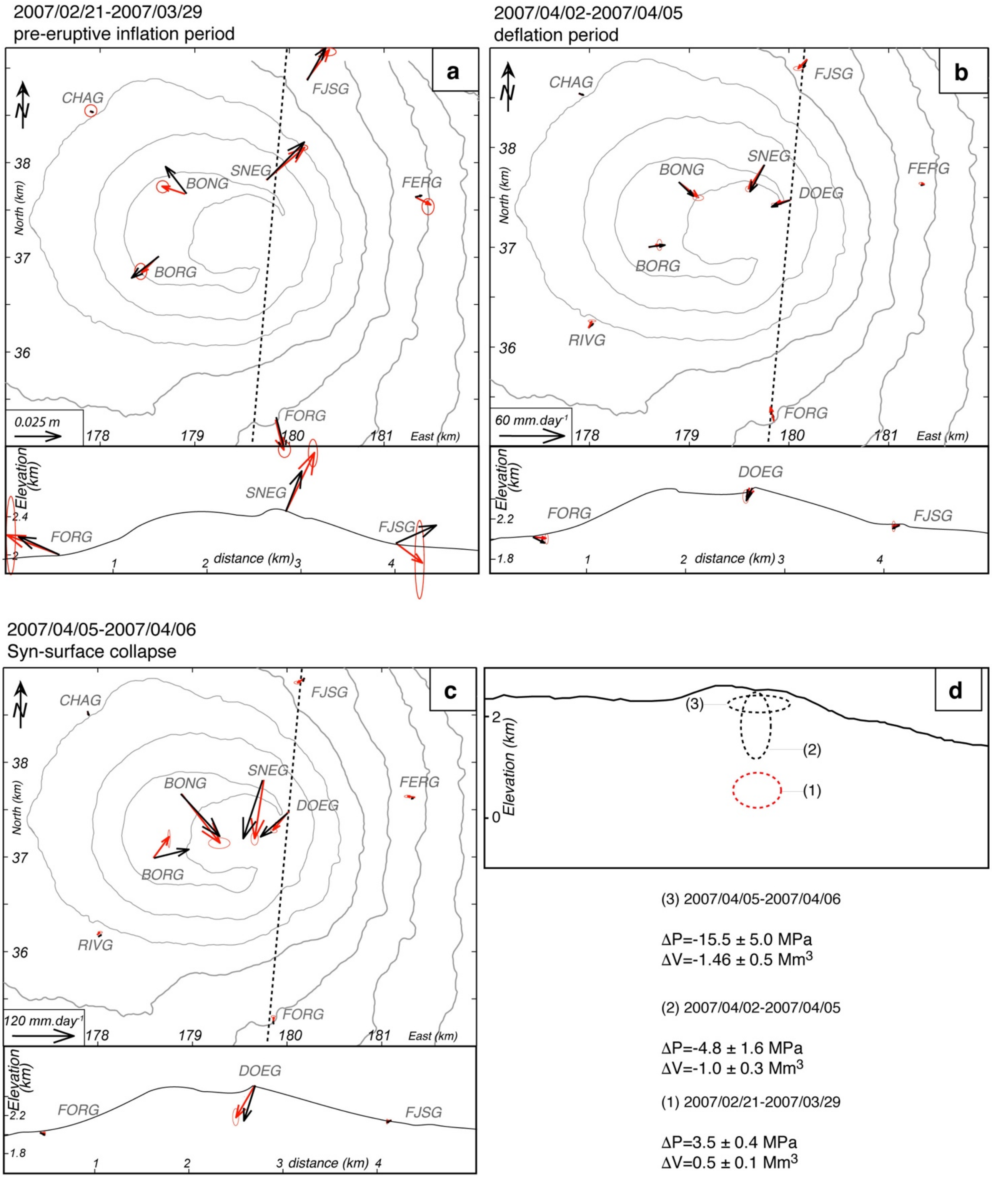

Figure S6. Results from inversion of the GPS data[25]. Figure modified from Peltier *et al.*[25]. (**a**) Observed (red) and synthetic (black) ground displacements for the pre-eruptive inflation period of February 21st-March 29th, 2007. Observed displacement rates (in red) and synthetic (in black) for the deflation period of April 2nd-5th (**b**) and for the syn-surface collapse of April

5th-6th (**c**). Uncertainty of each measurement is shown by an ellipse. Geographical coordinates are Gauss-Laborde Réunion kilometric coordinates (transverse Mercator). (**d**) Best location of the ellipsoidal source from the GPS inversion with the best modelled pressure and volume changes for the three periods represented in (a), (b), and (c). The red ellipse represents the likely location of the magma reservoir.

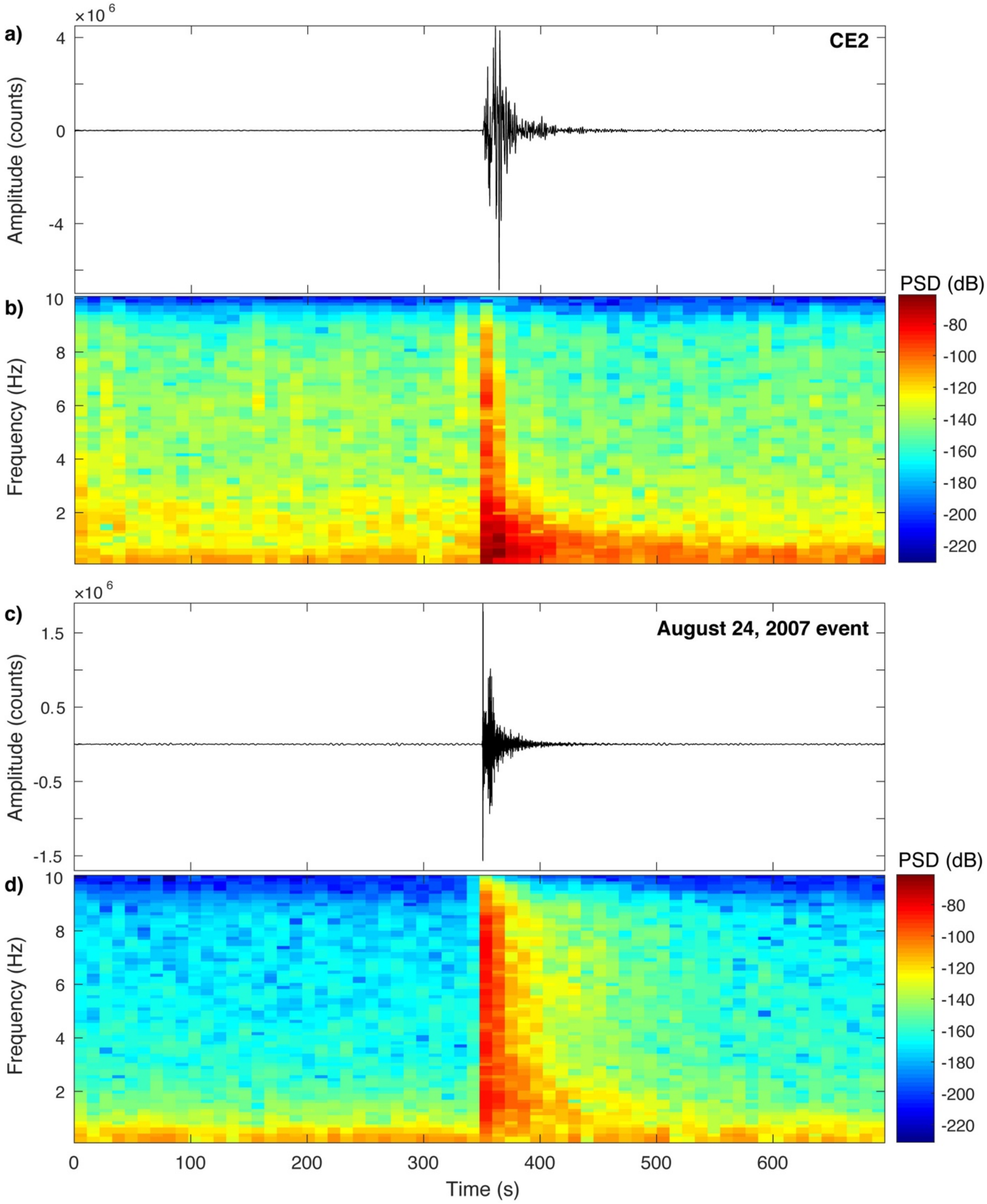

Figure S7. Comparison between the frequency content of the CE2 collapse event and a local event occurring on August 24, 2007 at 11:19. Spectrograms were computed from a 700 s long time series with a 8 s sliding time window. (**a**) Recording of the vertical component for the BH channel of RER station for the CE2 event with a duration magnitude $M_d \sim 3.5$. (**b**) Spectrogram of the CE2 event. The power spectral density (PSD) is represented in units of dB

related to 1 (m $s^{-1})^2$ Hz^{-1}. (**c**) Observed signal from the vertical component of RER station for a local event occurring on August 24, 2007 at 11:19. The duration magnitude of this event is M_d ~ 3.6. (**d**) The spectrogram of the August 24, 2007 event shows a higher frequency content than the spectrogram of the CE2 event. The PSD is represented in the same unit than in (b).

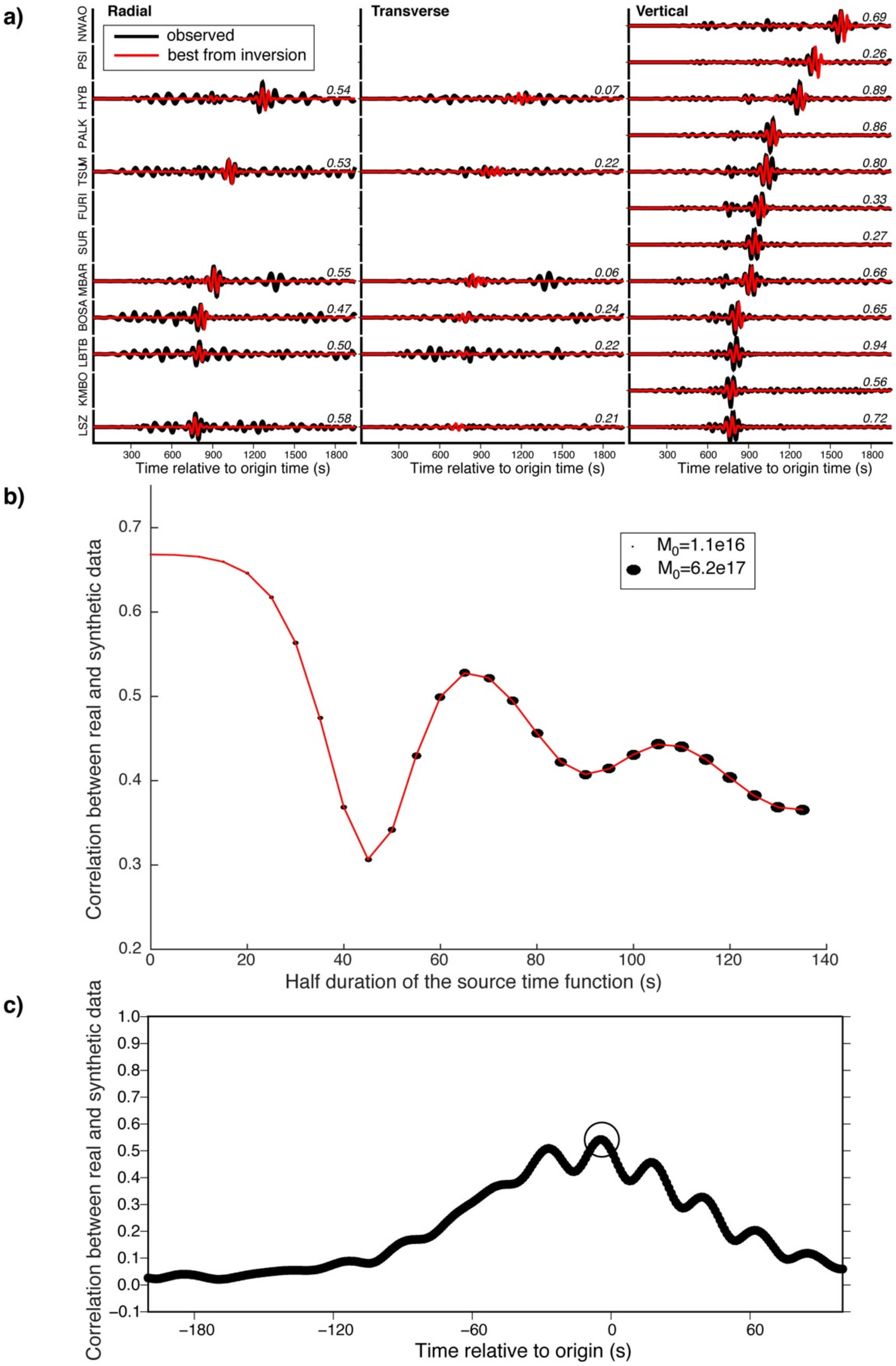

Figure S8. Results of the seismic moment tensor analysis. (**a**) Radial, transverse and vertical records in velocity filtered between 0.01 and 0.025 Hz (in grey) compared to the predicted seismograms (in red) obtained from the best inversion solution. The amplitudes are normalised by the maximum amplitude. The number shown in blue above each waveform is

the correlation coefficient between observed and synthetic data. (**b**) Results from the grid-search for the source time function duration (**c**) Time shift relative to the origin computed from the moment tensor inversion. The circle shows the location of the best solution.

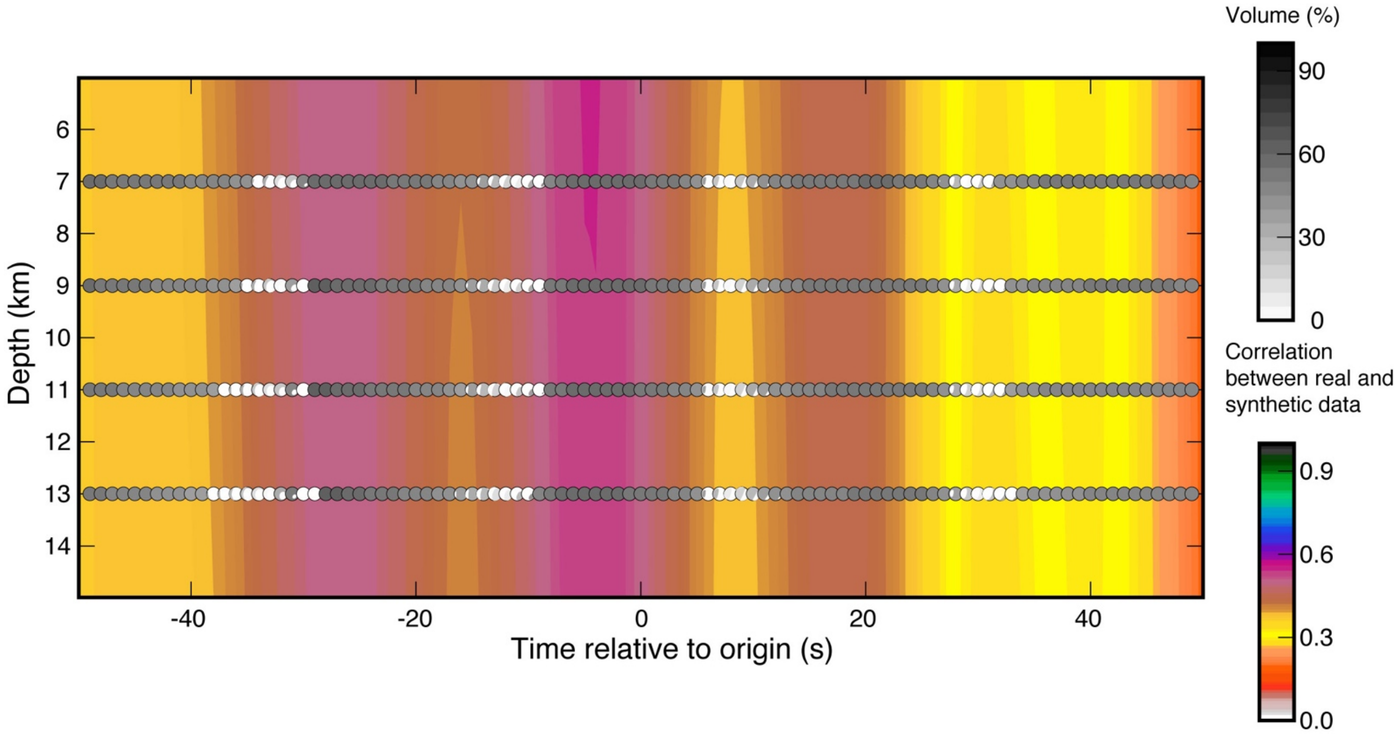

Figure S9. Results of the moment tensor inversion for time (relative to the origin) and depth. The background is color-coded for the correlation between real and synthetic data. Each focal mechanism is colored based on the volumetric percentage.

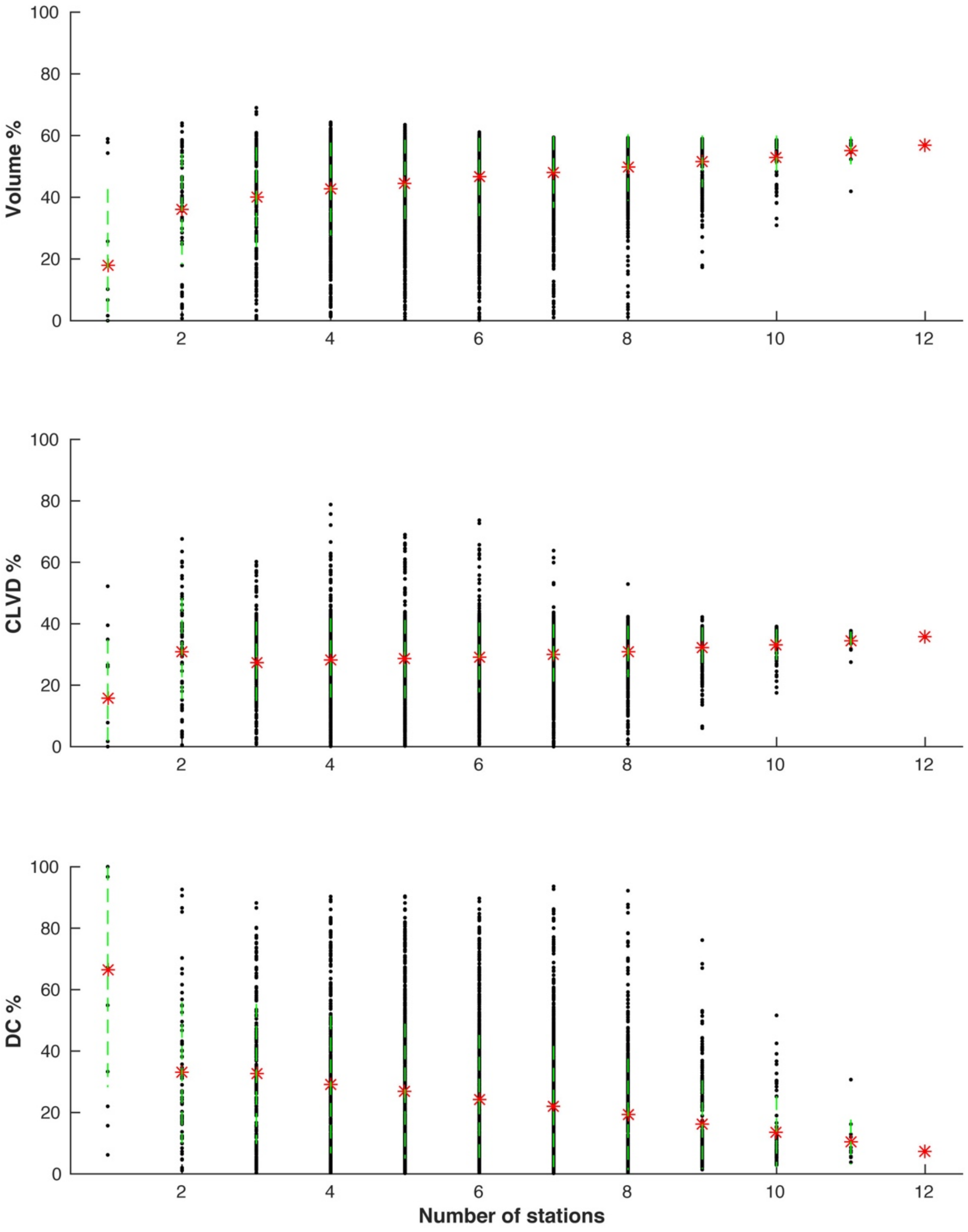

Figure S10. The Jackknife estimation method shows the stability of the strong isotropic and CLVD components. The percentage of the isotropic, CLVD and Double Couple (DC) components of the moment tensor is represented against the number of stations used in the inversion. The red star represents the mean percent isotropic or CLVD or DC component ± one standard deviation (i.e. the vertical green dashed line).

Supplementary method

Polarisation analysis of particle motions

The covariance matrix is equal to $(1/N_p)$ $(Y^T \cdot Y)$. Where N_p is the number of points in the time window and Y is a (N_p*3) centred matrix with the components (East, North, Vertical up) as its columns, and Y^T is the transpose of Y. Y is mean centred by column.

The degree of rectlinearity of the particle motion[74] is given by RL_{3D}=1-((λ_2+λ_3)/2λ_1), where λ_1, λ_2 and λ_3 are the eigenvalues of the covariance matrix and $\lambda_1 \geq \lambda_2 \geq \lambda_3$. RL_{3D} is 1.0 when $\lambda_2 = \lambda_3 = 0$ and $\lambda_1 \neq 0$ as expected for rectilinear particle motion and for pure body waves and it is close to 0 for an almost circular particle motion. Pure Rayleigh wave motion is expected to be elliptical.

We can also realize a principal component analysis (PCA) using the horizontal components only and compute the degree of rectilinearity of the particle motion[75-76] over the horizontal plane:

$$CpH = 1 - \frac{e_2}{e_1} \qquad \text{(S1)},$$

where e_2 and e_1 are the eigenvalues of the covariance matrix obtained from the two horizontal components and $e_2 \leq e_1$.

The apparent horizontal polarisation angle of the ground motion BAZ is:

$$BAZ = arctan\ (u_1/u_2) \qquad \text{(S2)},$$

where u_1 and u_2 are the Cartesian coordinates of u the eigenvector corresponding to the highest eigenvalue of the covariance matrix. u_1 corresponds to the coordinate relative to E and u_2 to the coordinate relative to N.

We can determine the longitudinal component L by $L = cos\ (BAZ+\pi)\ N + sin\ (BAZ+\pi)\ E$; where E and N are the horizontal components. Using the longitudinal and vertical

components, we can then determine a third covariance matrix and the degree of rectilinearity of the particle motion in the vertical plane:

$$CpZ = 1 - \frac{f_2}{f_1} \quad \text{(S3)},$$

where f_2 and f_1 are the eigenvalues of the covariance matrix determined using L and Z components and $f_2 \leq f_1$.

The vertical polarisation angle is obtained with:

$$VPA = \cos^{-1} (u_3) \quad \text{(S4)}$$

The uncertainty of the vertical polarisation angle in degrees is estimated from the approach proposed by D. Reymond[69, 70, 77]: E_{VPA}= (180/π) $arctan\ ((f_2/f_1)^{0.5})$, where f_1 and f_2 are the 2 eigenvalues obtained by the PCA in the vertical plane (from the longitudinal and vertical components). E_{BAZ} is defined as (180/π) $arctan[(e_2/e_1)^{0.5}]$ where e_1 and e_2 are respectively the eigenvalues obtained from the PCA applied only to the horizontal components.

Supplementary references

73. Genco, R. & Ripepe, M. Inflation-deflation cycles revealed by tilt and seismic records at Stromboli volcano. *Geophys. Res. Lett.* **37,** (2010).

74. Jurkevics, A. Polarization analysis of three-component array data. *Bull. seism. Soc. Am.* **78,** 1725–1743 (1988).

75. Flinn, E. A. Signal analysis using rectilinearity and direction of particle motion. *Proceedings of the IEEE*. **53,** 1874–1876 (1965).

76. Montalbetti, J. F. & Kanasewich, E. R. Enhancement of Teleseismic Body Phases with a Polarization Filter. *Geophys. J. R. astr. Soc.* **21,** 119–129 (1970).

77. Scholz, J.-R. *et al.* Orienting Ocean-Bottom Seismometers from P-wave and Rayleigh wave polarizations. *Geophys. J. Int.* **208,** 1277–1289 (2017). doi:10.1093/gji/ggw426.

Supplementary Table 1. Chronological list of all 53 events reported in this study.

Number	Day (Year/Month/Day)	Day of year	Hour	Minutes	Seconds	M_S	σ	*CC*
E1	2007/03/30	89	16	31	25			
E2	2007/04/05	95	0	47	56			0.79
E3	2007/04/05	95	1	3	49			0.74
E4	2007/04/05	95	13	47	46			0.74
E5	2007/04/05	95	16	19	8			0.83
CE1	2007/04/05	95	20	48	43	4.8	0.2	
CE2	2007/04/05	95	22	33	28	4.1	0.2	0.98
CE3	2007/04/06	96	0	39	33	4.1	0.2	0.97
CE4	2007/04/06	96	2	21	51	4.1	0.2	0.98
CE5	2007/04/06	96	3	48	40	4.1	0.2	0.98
CE6	2007/04/06	96	5	3	43	4.1	0.2	0.98
CE7	2007/04/06	96	6	9	4	4.0	0.3	0.98
CE8	2007/04/06	96	7	7	35	4.1	0.1	0.98
CE9	2007/04/06	96	8	0	35	4.1	0.1	0.97
CE10	2007/04/06	96	8	52	40	4.0	0.1	0.97
CE11	2007/04/06	96	9	36	37	4.0	0.1	0.94
CE12	2007/04/06	96	10	14	58	4.0	0.1	0.95
CE13	2007/04/06	96	10	56	49	4.0	0.1	0.97
CE14	2007/04/06	96	11	31	40	3.8	0.1	0.94
CE15	2007/04/06	96	12	5	15	3.8	0.1	0.93
CE16	2007/04/06	96	12	36	40	3.8	0.1	0.92
CE17	2007/04/06	96	13	8	33	3.9	0.2	0.88
CE18	2007/04/06	96	13	38	36	3.8	0.1	0.91
CE19	2007/04/06	96	14	6	28	3.8	0.1	0.89
CE20	2007/04/06	96	14	35	55	3.8	0.2	0.86
CE21	2007/04/06	96	15	6	0	3.7	0.1	0.94
CE22	2007/04/06	96	15	38	36	3.8	0.2	0.89
CE23	2007/04/06	96	16	8	46	3.7	0.2	0.90
CE24	2007/04/06	96	16	37	3	3.6	0.1	0.76
CE25	2007/04/06	96	17	8	37	3.7	0.1	0.91
CE26	2007/04/06	96	17	40	39	3.6	0.2	0.86
CE27	2007/04/06	96	18	14	15	3.7	0.2	0.79
CE28	2007/04/06	96	18	47	20	3.6	0.1	0.87
CE29	2007/04/06	96	19	29	36			0.82
CE30	2007/04/06	96	20	4	1	3.7	0.1	0.71
CE31	2007/04/06	96	20	38	37			0.71
CE32	2007/04/06	96	21	10	24			0.70
CE33	2007/04/06	96	21	50	47	3.7	0.1	0.79
CE34	2007/04/06	96	22	29	29	3.6	0.2	0.63
CE35	2007/04/06	96	23	11	6	3.7	0.1	0.83
CE36	2007/04/06	96	23	56	51	3.6	0.2	0.73
CE37	2007/04/07	97	0	51	33	3.7	0.2	0.72
CE38	2007/04/07	97	3	12	42	3.7	0.2	0.83
CE39	2007/04/07	97	4	58	7	3.6	0.1	0.75

CE40	2007/04/07	97	6	24	20			0.69
CE41	2007/04/07	97	8	19	1			0.86
CE42	2007/04/07	97	11	3	58			0.85
CE43	2007/04/07	97	15	9	33	3.8	0.1	0.76
CE44	2007/04/08	98	0	7	20	3.9	0.1	0.85
CE45	2007/04/09	99	2	45	25	3.9	0.2	0.93
CE46	2007/04/09	99	13	34	52	4.0	0.2	0.98
CE47	2007/04/12	102	11	5	47	4.2	0.3	0.96
CE48	2007/04/14	104	0	19	33	4.2	0.2	0.96

E1 was accompanied with a ULP signal. 4 events (E2 to E5) were observed with both VLP and ULP signals before the caldera collapse. Some 48 events (CE1 to CE48) showed both VLP and ULP signals during the Dolomieu Caldera collapse. Starting time is determined on BH records from the RER seismometer. The surface wave magnitude (M_S) value is also included and the standard deviation σ of each M_S estimate. CC is the cross-correlation coefficient of the VLP signals of CE1 with those of the other events.

Supplementary Table 2. Results from polarisation analysis of ULP seismic signals recorded before and during the caldera collapse (E1 to CE48).

N	**Day** (Year/Month/Day)	*BAZ* (°)	E_{BAZ} (°)	*VPA* (°)	E_{VPA} (°)	*CpH*	*CpZ*	*Depth (km)*	*ΔD (km)*
E1	2007/03/30	182.5	5.3	96.3	3.4	1.0	1.0	-0.1	0.5
E2	2007/04/05	183.9	5.2	96.5	6.3	1.0	1.0	-0.1	1.0
E5	2007/04/05	188.3	13.9	98.7	11.2	0.9	1.0	-0.5	1.7
CE1	2007/04/05	188.7	6.9	*NC*	32.5	1.0	0.6	*NC*	*NC*
CE2	2007/04/05	186.9	8.9	103.7	18.5	1.0	0.9	-1.3	3.1
CE3	2007/04/06	186.9	9.5	106.5	19.0	1.0	0.9	-1.7	3.2
CE4	2007/04/06	187.5	9.0	104.5	17.6	1.0	0.9	-1.4	2.9
CE5	2007/04/06	187.3	9.2	103.3	16.6	1.0	0.9	-1.2	2.7
CE6	2007/04/06	187.1	9.0	102.8	15.8	1.0	0.9	-1.1	2.6
CE7	2007/04/06	187.0	9.7	103.0	15.4	1.0	0.9	-1.1	2.5
CE8	2007/04/06	187.6	8.9	101.1	13.9	1.0	0.9	-0.9	2.2
CE9	2007/04/06	186.5	9.5	101.4	14.0	1.0	0.9	-0.9	2.2
CE10	2007/04/06	186.0	9.8	101.9	14.1	1.0	0.9	-1.0	2.3
CE11	2007/04/06	187.5	9.1	99.7	12.1	1.0	1.0	-0.6	1.9
CE12	2007/04/06	187.1	9.8	99.9	13.5	1.0	0.9	-0.7	2.1
CE13	2007/04/06	186.9	9.3	100.1	12.7	1.0	0.9	-0.7	2.0
CE14	2007/04/06	186.8	9.7	100.2	13.0	1.0	0.9	-0.7	2.1
CE15	2007/04/06	186.8	9.1	102.5	15.3	1.0	0.9	-1.1	2.5
CE16	2007/04/06	187.3	9.1	99.5	11.8	1.0	1.0	-0.6	1.8
CE17	2007/04/06	186.6	9.2	99.8	13.5	1.0	0.9	-0.7	2.1
CE18	2007/04/06	187.5	8.6	102.7	16.4	1.0	0.9	-1.1	2.7
CE19	2007/04/06	186.9	9.6	100.5	13.3	1.0	0.9	-0.8	2.1
CE20	2007/04/06	187.5	9.2	100.0	11.8	1.0	1.0	-0.7	1.9
CE21	2007/04/06	187.5	9.2	98.8	13.5	1.0	0.9	-0.5	2.1
CE22	2007/04/06	187.0	9.0	101.7	12.2	1.0	1.0	-1.0	1.9
CE23	2007/04/06	187.6	8.7	101.5	13.9	1.0	0.9	-0.9	2.2
CE24	2007/04/06	186.7	9.1	103.7	16.6	1.0	0.9	-1.3	2.7
CE25	2007/04/06	186.3	9.0	99.6	12.5	1.0	1.0	-0.6	2.0
CE26	2007/04/06	186.9	8.7	102.4	19.4	1.0	0.9	-1.1	3.2
CE27	2007/04/06	186.8	8.9	99.6	13.0	1.0	0.9	-0.6	2.1
CE28	2007/04/06	187.2	9.3	99.6	13.0	1.0	0.9	-0.6	2.0
CE29	2007/04/06	187.9	9.5	100.0	12.0	1.0	1.0	-0.7	1.9
CE30	2007/04/06	186.6	9.7	100.5	17.1	1.0	0.9	-0.8	2.7
CE31	2007/04/06	187.1	9.2	101.2	14.3	1.0	0.9	-0.9	2.3
CE32	2007/04/06	187.0	9.4	101.1	15.5	1.0	0.9	-0.9	2.5
CE33	2007/04/06	187.6	9.7	100.8	13.8	1.0	0.9	-0.8	2.2
CE34	2007/04/06	187.0	8.7	100.4	14.4	1.0	0.9	-0.8	2.3
CE35	2007/04/06	187.2	9.0	100.4	13.0	1.0	0.9	-0.8	2.1
CE36	2007/04/06	187.1	8.8	99.4	11.7	1.0	1.0	-0.6	1.8
CE37	2007/04/07	187.9	9.2	100.4	13.6	1.0	0.9	-0.7	2.2
CE38	2007/04/07	188.2	8.7	99.9	12.3	1.0	1.0	-0.7	1.9
CE39	2007/04/07	188.1	8.1	101.8	14.0	1.0	0.9	-1.0	2.2
CE40	2007/04/07	188.0	8.6	100.5	14.3	1.0	0.9	-0.8	2.3
CE41	2007/04/07	188.4	8.6	100.9	14.4	1.0	0.9	-0.8	2.3

CE42	2007/04/07	189.4	8.1	100.2	12.7	1.0	0.9	-0.7	2.0
CE43	2007/04/07	188.7	7.9	99.7	12.3	1.0	1.0	-0.6	1.9
CE44	2007/04/08	188.9	7.9	99.7	12.2	1.0	1.0	-0.6	1.9
CE45	2007/04/09	189.4	7.3	99.6	12.0	1.0	1.0	-0.6	1.9
CE46	2007/04/09	188.7	8.0	100.3	12.8	1.0	0.9	-0.7	2.0
CE47	2007/04/12	189.7	7.5	102.3	15.0	1.0	0.9	-1.0	2.4
CE48	2007/04/14	189.0	7.9	102.1	15.2	1.0	0.9	-1.0	2.5

N is the event number, *BAZ* indicates the apparent horizontal polarisation angle and *VPA* is the vertical polarisation angle. *NC* is for not constrained *VPA* due to the uncertainty on the parameter. E_{BAZ} and E_{VPA} are estimations of the uncertainties of the apparent horizontal and vertical polarisation angles (see Supplementary information). *CpH* and *CpZ* are the degree of rectilinearity of the particle motion in the horizontal plane and in the vertical plane respectively (see Supplementary information). *Depth* is the estimated source depth and ΔD is its uncertainty. E3 and E4 were not considered because of low signal-to-noise ratio: < 2.0.
